# Crosstalk Between Chaperone-Mediated Protein Disaggregation and Proteolytic Pathways in Aging and Disease

**DOI:** 10.3389/fnagi.2019.00009

**Published:** 2019-01-29

**Authors:** Diogo R. Feleciano, Katrin Juenemann, Manuel Iburg, Inês C. Brás, Carina I. Holmberg, Janine Kirstein

**Affiliations:** ^1^Leibniz Institute for Molecular Pharmacology (FMP) im Forschungsverbund Berlin, Berlin, Germany; ^2^Department of Experimental Neurodegeneration, University Medical Center Goettingen, Goettingen, Germany; ^3^Research Programs Unit, Translational Cancer Biology Program, and Medicum, Biochemistry and Developmental Biology, University of Helsinki, Helsinki, Finland

**Keywords:** *C. elegans*, chaperone, aging, disaggregation, proteolysis, proteostasis

## Abstract

A functional protein quality control machinery is crucial to maintain cellular and organismal physiology. Perturbation in the protein homeostasis network can lead to the formation of misfolded and aggregated proteins that are a hallmark of protein conformational disorders and aging. Protein aggregation is counteracted by the action of chaperones that can resolubilize aggregated proteins. An alternative protein aggregation clearance strategy is the elimination by proteolysis employing the ubiquitin proteasome system (UPS) or autophagy. Little is known how these three protein aggregate clearance strategies are regulated and coordinated in an organism with the progression of aging or upon expression of disease-associated proteins. To unravel the crosstalk between the protein aggregate clearance options, we investigated how autophagy and the UPS respond to perturbations in protein disaggregation capacity. We found that autophagy is induced as a potential compensatory mechanism, whereas the UPS exhibits reduced capacity upon depletion of disaggregating chaperones in *C. elegans* and HEK293 cells. The expression of amyloid proteins Aβ_3–42_ and Q_40_ result in an impairment of autophagy as well as the UPS within the same and even across tissues. Our data indicate a tight coordination between the different nodes of the proteostasis network (PN) with the progression of aging and upon imbalances of the capacity of each clearance mechanism.

## Introduction

The maintenance of a functional proteome is critical for the cellular and organismal physiology. The cell is equipped with a powerful protein homeostasis (proteostasis) network (PN) that safeguards the proteome. The PN is composed of molecular chaperones and degradation pathways, such as autophagy and the ubiquitin-proteasome system (UPS). Molecular chaperones constitute a large and diverse protein family that plays a key role in the maintenance of proteostasis. Chaperones ensure the native state of proteins by assisting *de novo* folding, preventing misfolding and by protein refolding. Furthermore, specific chaperones can also reverse protein aggregation (Hartl et al., 2011). The recently described HSP70/110/J chaperone complex can suppress aggregation and promote disaggregation of amorphous as well as disease-associated amyloid aggregates such as α-synuclein and mutant huntingtin (Rampelt et al., 2012; Gao et al., 2015; Nillegoda et al., 2015; Scior et al., 2018).

The UPS is the main clearance pathway for the degradation of un- and misfolded proteins. Substrates for the UPS are poly-ubiquitinated *via* a cascade reaction conducted by E1, E2 and E3 ligases. Subsequently, the ubiquitinated proteins are targeted to, unfolded and degraded by the 26S proteasome in an energy-dependent process (Hershko et al., 1980). The formation of specific poly-ubiquitin linkages allows the substrate recognition by the 19S regulatory particle and subsequent degradation by the 20S core particle into small peptides. The UPS is a key component of the PN, and its function is indispensable for the efficient removal of toxic proteins. Neurodegenerative diseases-related proteins such as the N-terminal fragment of mHtt can also be degraded by the proteasome (Juenemann et al., 2013). Despite this observation, the poly-ubiquitin tagging and the degradation rates are not sufficient to prevent an accumulation of aggregated mHtt that ultimately leads to proteasome dysfunction (Bence et al., 2001; Holmberg et al., 2004).

Autophagy is a crucial mechanism that allows the cell to eliminate specific misfolded and aggregated proteins, dysfunctional organelles or pathogens (Dunn, 1990; Shintani and Klionsky, 2004). In a cascade of events a autophagosome is formed and fuses with a lysosome that subsequently leads to the breakdown of the cargo (Mizushima et al., 2010). This process can be selective and non-selective, but mostly relies on the recognition of ubiquitinated organelles/proteins by an autophagic adaptor, SQST-1/p62 or NBR-1 that binds to LC3-II (LGG-1 in *C. elegans*), which allows the engulfment of the toxic species and membrane closure. The autophagy pathway has been shown to eliminate disease-associated proteins such as Aβ, α-synuclein or mHtt (Ravikumar et al., 2002; Webb et al., 2003; Yu et al., 2004). Moreover, neurodegenerative disease models are known to exhibit a dysfunctional autophagic flux and it has also been shown that the polyglutamine region of disease-related proteins directly affects autophagy (Menzies et al., 2015; Ashkenazi et al., 2017).

With the progression of aging, the accumulation of misfolded and toxic proteins poses a challenge to the cellular PN. None of the clearance pathways is sufficient to prevent an accumulation of aggregated protein species. Many efforts have been made to characterize how protein quality control mechanisms are regulated at young age and with aging as well as in neurodegenerative diseases. Nevertheless, it remains unknown if and how the proteolytic clearance of protein aggregates and the chaperone-mediated disaggregation are coordinated to enable the cell and the organism to cope with proteotoxic protein aggregates especially during aging (Rubinsztein et al., 2011; Kaushik and Cuervo, 2015).

To unravel the crosstalk between the different protein aggregate clearance options, we investigated how autophagy and UPS respond to the expression of disease-associated proteins and during aging when the chaperone capacity of those chaperones that constitute the disaggregase complex, is compromised. Here, we show that in wt animals autophagy is reduced with aging, whereas the UPS exhibits a tissue specific activity that shows elevated as well as reduced capacity with the progression of aging. Depletion of disaggregating chaperones leads to an activation of autophagy that probably serves as compensatory mechanism to rebalance proteostasis. Notably, this compensatory effect of autophagy is diminished as aging progresses beyond the reproductive phase of *C. elegans*. Interestingly, the proteasomal activity and 20S levels are reduced upon knockdown of the chaperones. Thus, the UPS responds in a complete opposite manner to autophagy when chaperone-mediated resolubilization of protein aggregates is compromised. Finally, we show that expression of aggregation-prone and disease-associated Aβ_3–42_ and polyglutamine (Q_40_) proteins leads to an impairment of both proteolytic pathways and perturb proteasome function within the same, but surprisingly also across tissues. Our study further elucidates the interplay of the different protein aggregate clearance mechanisms of the PN in aging and disease on the cellular and organismal level.

## Materials and Methods

### *C. elegans* Strains and Maintenance and RNAi

Nematode strains used in this study: Bristol strain N2 (wild type, wt), DA2123 (adIs2122 (lgg-1p::GFP::lgg-1 + rol-6(su1006))), RT476 (wIs170 (vha6p::GFP::rab-7 + Cbr-unc-119(+))), MAH240 (sqIs17 (hlh-30p::hlh-30::GFP + rol-6(su1006))), YD3 (xzEx3(Punc-54::UbG76V::Dendra2)), YD12 (xzEx12(PF25B3.3::UbG76V::Dendra2)), CL2006 (dvIs2 (pCL12(unc-54:hu-Aβ_1–42_) + pRF4)), CL2355 (smg-1ts (cc546); snb-1::Aβ_1–42_::long 3′-UTR), AM141 (rmIs133 (*unc54*p::Q_40_::YFP)), AM1066 (rmIs350 (unc-54p::Q_40_::RFP)), AM101 (rmIs110 (F25B3.3p::Q_40_::YFP)), AM44 (rmIs190 (F25B3.3p::Q_67_::CFP)), HZ589 (bpIs151 (sqst-1p::sqst-1::GFP + unc-76(+))), DA2123/CL2006, DA2123/AM1066, HZ589/CL2006, YD3/CL2006, YD3/CL2355, YD12/CL2006 and YD12/CL2355. Nematodes were grown on NGM plates seeded with *E. coli* OP50 at 20°C. Synchronized animals were placed onto RNAi plates seeded with *E. coli* expressing ds RNA against *hsp-1*, *hsp-110*, *dnj-13, hsp-16.41, hsp-17* or *pas-5* or the empty vector L4440 as a control. The animals were analyzed in the indicated days of life. The *hsp-16.41* and* hsp-17* RNAi constructs were generated by cloning the respective cDNA into the vector L4440 (Timmons and Fire, 1998) using the primers: *hsp-16.41*: ataagaatggtaccatTTTTTCCGATAATATTGGGGAG and cagtcaaagcttTCCATGTTCCGATTTTGTTTC; *hsp-17*: ataagaatggtaccatGGACACCGAGTAGGAGATGC and cagtcaaagcttTCAGTCTTCTCGTTATGCTTTCC.

### Cell Culture and Transfection

HEK293 cells were maintained in DMEM supplemented with 10% fetal calf serum, 1 mM glutamine, 100 U/ml penicillin, and 100 μg/ml streptomycin. Cells were transfected with jetPRIME according to the manufacturer’s instructions. For siRNA mediated knockdown siGENOME SMARTpool DNAJB1 (M-012735-02), HSPA4 (M-012636-02) and HSPA8 (M-017609-01) siRNA were purchased from Dharmacon and transfected in a final concentration of 50 nM. Non-targeting siRNA (Dharmacon siGENOME Non-Targeting siRNA Pool #1 D-001206-13; for FlucDM-GFP experiments—Non-Targeting siRNA #3 D-001210-03) was used as negative control. Cells were transfected twice with siRNA (24 and 48 h). For overexpression of UbG76V-GFP, cells were co-transfected with 2 μg DNA 24 h prior harvest. Cells were treated with 200 nM Bafilomycin A1 (BafA1; Sigma #B1793) or 20 μM MG-132 (Sigma #M7449) 4 h before harvest. After 48 h cells were harvested with the respective lysis buffer (50 mM Tris/HCl pH 7.4, 150 mM NaCl, 1 mM EDTA, 1% Triton-X100, complete mini protease inhibitor cocktail (Roche)). For overexpression experiments, UbG76V-GFP cloned into the pEGFP-N1 vector was used. For transient expression of GFP, HttExon1Q_25_-GFP and HttExon1Q_97_-GFP HEK293 cells were transfected with 2 μg Plasmid-DNA 24 h prior cell harvest.

### Live Cell Imaging and Biochemical Analysis

HEK293 cells expressing FlucDM-GFP were seeded in fluorobrite DMEM medium in an 8-well glass bottom plate (Ibidi #80827) coated with poly L-lysine (Sigma #P5899). Cells were treated with siRNA and imaged with a 40x objective for 48 h in a CSU microscope (Nikon) supplied with 5% CO_2_ at 37°C. For luminescence activity experiments, cells were seeded in 24 well-plates format and treated with siRNA or MG-132 and incubated with Steady-Glo Luciferase mix (Promega #E2510) for the indicated time points in the dark at room temperature for 10 min and the luminescence signal was acquired for 1 s in a plate reader (Tecan Safire).

### Protein Isolation

*C. elegans*—50 animals were washed in M9 and the pellets frozen in liquid nitrogen. 2× Laemmli loading buffer (50 mM Tris-HCl pH 6.8, 2% SDS, 15% glycerol, 100 mM DTT, 0.1% bromophenol-blue) was added to the samples and incubated with shaking for 10 min at 100°C, centrifuged for 5 min at 5,000 rpm and prior to SDS-PAGE.

HEK 293 cells—cells were harvest in lysis buffer (50 mM Tris/HCl pH 7.4, 150 mM NaCl, 1 mM EDTA, 1% Triton-X100, complete mini protease inhibitor cocktail (Roche)) and incubated for 30 min on ice. Prior to SDS-PAGE, 30 μg of protein were boiled for 5 min with 6× Laemmli loading buffer (350 mM Tris-HC pH 6.8, 10% SDS, 30% glycerol, 6% β-Mercaptoethanol, 0.3% bromophenol-blue).

### Antibodies

The protein lysates of *C. elegans* or HEK293 cells were separated in an SDS-PAGE and transferred to a PVDF membrane (Trans-blot Turbo system, Bio-Rad). In this study the following antibodies were used: anti-α-tubulin (1:2,000, Sigma #T5168), anti-β-actin (1:2,000, Santa Cruz), anti-HSP-1/ HSP-110/DNJ-13 (1:5,000, (Scior et al., 2018)), anti-DNAJB1 (1:1,000, Proteintech #13174-1-AP), anti-20S α-subunits (1:1,000, Enzo #MCP231), anti-LGG-1/SQST-1/p62 (1:1,000), anti-HSP-17 (1:2,500) and anti-LC3 (1:1,000, Abcam #ab48394), anti-mouse/rabbit HRP (1:10,000/1:15,000, Thermo Fisher #31444/#31460) or anti-mouse-Cy3 antibody (1:10,000, Licor #926-68072). LGG-1 and SQST-1/p62 antibodies were generated by immunizing rabbits with a synthetic peptide. LGG-1: PKSKLHDLDKKKYL; SQST-1/p62: AVPKPAQEPRIPPSPTSALPPPQFFN (Pineda, Germany). HSP-17 antibody was generated by immunizing rabbits with the purified protein (Pineda, Germany).

### Compound Treatment

Nematodes were treated with 0.2% DMSO (Carl Roth #A994), 200 μM of rapamycin (Sigma #R0395), 10 mM CQ (Cell Signaling #14774) or 2 μM Lysotracker-Red DND-99 (Thermo Fisher #L7528) in M9 buffer supplement with OP50 for 18 h in a 96-well plate at 20°C.

### Confocal Imaging

Animals were mounted onto glass slides with 2% agarose (Bio and Sell) in M9. The strain MAH240 and the LysoTracker-Red stained animals were imaged (with 0.1% NaN3 (Carl Roth #4221)) in an EPI-TIRF microscope (Nikon) with a 4× objective. The RT476, DA2123 (with 2 mM levamisole (AppliChem #A4341)) and YD3/YD12 (the experimental set-up is described below) strains were imaged in a confocal microscope LSM710 (Zeiss) with 10× or 20× objectives.

**Figure 1:**
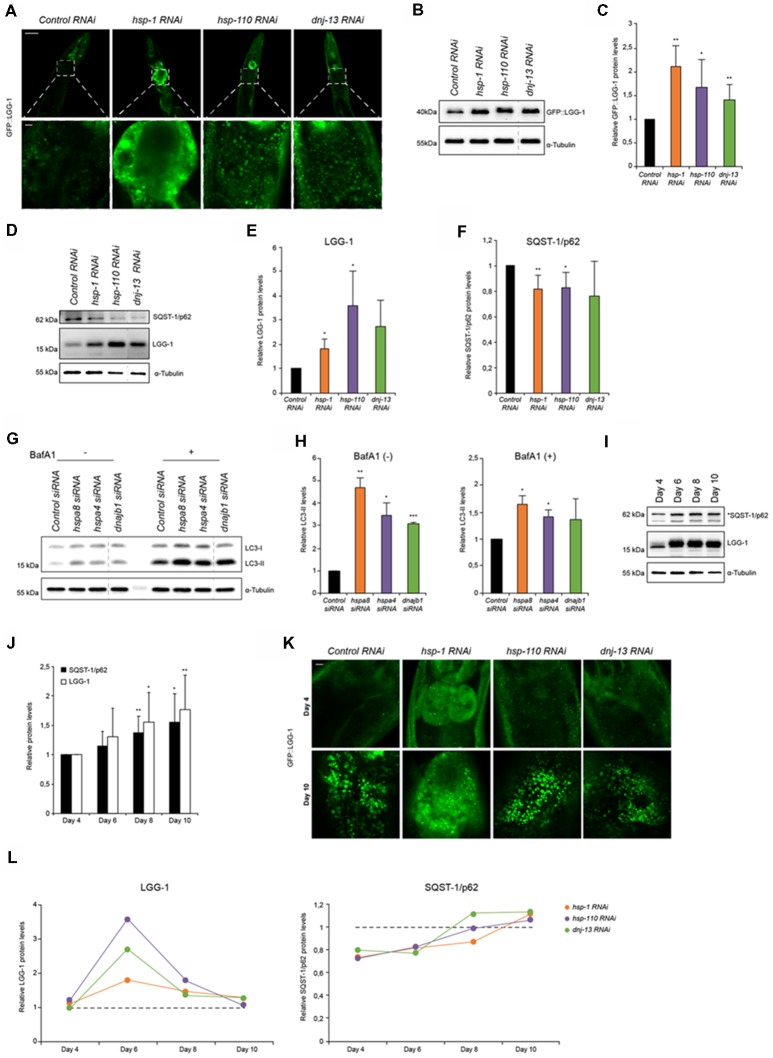
Autophagy is induced when chaperone disaggregase capacity is compromised. **(A)** Fluorescent images of GFP::LGG-1 animals (day 6) subjected to RNAi to deplete *hsp-1*, *hsp-110* and *dnj-13*. Upper panel shows the head/intestinal region of the animals and the lower panel is a magnification of the indicated regions. Scale bars: 50 μm (upper) and 5 μm (lower panel). **(B,C)** Immunoblotting of GFP::LGG-1 RNAi treated animals (as in **A**). The error bars represent the standard deviation (SD) from a minimum of four independent experiments **(B)**. The dashed lines indicate that some lanes were removed from the immunoblot. **(D–F)** Relative protein levels of LGG-1 and SQST-1/p62 from RNAi treated wild type (wt) animals (day 6). The error bars represent the SD of a minimum of three independent experiments. **(G,H)** Immunoblotting of siRNA transfected HEK293 cells treated with 200 nM Bafilomycin A1 (BafA1). LC3-II levels were normalized to α-tubulin levels and then to siRNA control **(H)**. The error bars represent the SD of two independent experiments. **(I,J)** LGG-1 and SQST-1/p62 levels of nematodes of 4, 6, 8 and 10 days of age. Depicted are the relative LGG-1 and SQST-1/p62 levels normalized day 4 **(J)**. The error bars represent the SD of a minimum of five independent experiments. **(K)** Fluorescent images of RNAi treated GFP::LGG-1 animals. The fluorescent images are a magnification of the intestinal region and were acquired at day 4 (upper) and day 10 (lower panel). Scale bars: 5 μm. **(L)** Relative LGG-1 and SQST-1/p62 levels of day 4, 6, 8 and 10 of RNAi treated animals. The values were normalized to the control RNAi of the respective day. Graphs represent the average values from a minimum of two independent experiments.

Brightness and contrast of images were adjusted equally between the different conditions. Due to high fluorescence intensity, some images were edited separately: in Figure 1—*hsp-1* RNAi images have less brightness adjustments due to the high fluorescence; in Figure 1K—day 10 images have less brightness adjustments compared to day 4.

Fiji ImageJ software (employing the “Find Maxima” function) was used to determine GFP::LGG-1 and SQST-1/p62::GFP puncta. Fluorescent intensities of DA2123/CL2006, DA2123/AM1066, RT476 and LysoTracker-Red stained animals were determined for each animal (fluorescence was normalized to area of animal) with Fiji ImageJ software.

### Proteasome Activity Assays

In *C. elegans*, the YD3 and YD12 strains were used to measure the degradation rates of UbG76V::Dendra2. The photo-conversion of single-cells in the strains YD3 and YD12 was performed as described previously (Hamer et al., 2010). Briefly, animals were mounted on 3% agarose pads with 0.5 mM levamisole. Photo-conversion and imaging were performed using a LSM710 microscope. After imaging, worms were recovered in the respective feeding plate and imaged 3 (YD12) or 24 h (YD3) later. The UbG76V::Dendra2 fluorescence was quantified with Fiji ImageJ software.

In HEK293 cell, the UbG76V-GFP levels were measured by western blot.

Chymotrypsin-like activity was analyzed as previously described (Kisselev and Goldberg, 2005). Briefly, 25 μg (*C. elegans*) or 10 μg (HEK293 cells) of protein were incubated with 100 μM of Suc-LLVY-AMC (Enzo, #BML-P802-0005) in a 96well plate (Corning #3615). As a positive control, 50 μM (*C. elegans*) or 20 μM (HEK293 cells) of MG-132 was incubated with the protein lysate for 30 min on ice before the addition of Suc-LLVY-AMC. A microplate reader (Tecan Safire) was used to monitor the fluorescence (380ex/460em) with measurements each 5 min for 2 h at 25°C (*C. elegans*) or 37°C (HEK293 cells). Each condition was done in duplicates. The slope (in the linear phase) was used to calculate the relative activities and then normalized to the control condition.

### qRT-PCR

Quantitative RT-PCR was performed as described previously (Kirstein et al., 2017). The sequences of the primers used in this study were the following:

*cdc-42*:

5′-AAACTTGTCTCCTGATCAGCT-3′ (forward)
5′-TACTGTG ACGGCGTAATTGT-3′ (reverse)

*rpn-6.1*:

5′-ATGAAAAAGTGTCCGCCCTC-3′ (forward)
5′-AGTTTTGCGGACAGAAGACT-3′ (reverse)

*pas-5*:

5′-CGCTGCTGAGAAAAGATCGA-3′ (forward)
5′-GGCAATCAAACCTGCGAATG-3′ (reverse)

*pbs-5*:

5′-GCCACTTATCGGGATTCTGG-3′ (forward)
5′-CTCGTACCACAGCTTGCTC-3′ (reverse)

### Statistical Analyses

The data was analyzed with ANOVA to test for statistical significance. We considered *p* values < 0.05 significant: *p* < 0.05 (*), < 0.01(**), < 0.001 (***).

## Results

### Autophagy Is Induced When Chaperone Capacity Is Compromised

Disaggregation of protein aggregates in higher eukaryotes is mediated by a trimeric chaperone complex composed of members of Hsp70, Hsp110 and J-proteins (Rampelt et al., 2012; Kirstein et al., 2017). To elucidate the impact of the disaggregase complex HSP70/110/J on the clearance of protein aggregates by the proteolytic pathways, autophagy and the UPS, we performed RNAi-mediated knockdown of a selected set of chaperones. The chaperones HSP-1 (Hsc70), HSP-110 and DNJ-13 constitute a powerful protein disaggregase in *C. elegans* enabling the disaggregation of e.g., mutant HttExon1Q_48_ (Rampelt et al., 2012; Kirstein et al., 2017) and amorphous aggregates formed by e.g., luciferase (Nillegoda et al., 2015; Kirstein et al., 2017). The knockdown efficiencies by RNAi treatment were analyzed by western blot and revealed remaining chaperone protein levels of 20%–40% for HSP-1, HSP-110 and DNJ-13 (Supplementary Figures S1A,B).

To study the autophagy pathway, we used a well-established autophagy reporter strain that encodes the LGG-1 protein tagged with GFP expressed ubiquitously in the animal (Melendez et al., 2003). This reporter enables the visualization of autophagic structures by light microscopy. The wt animal displays as young adult (day 6) a low basal level of autophagic vesicles in the head and intestine. Depletion of the chaperones led to a pronounced accumulation of autophagosomal structures mainly in the intestine posterior to the pharynx (Figure 1A; Supplementary Figures S1E,F). The increased number of GFP::LGG-1 positive structures detected by confocal microscopy could also be confirmed by western blot using an anti-GFP antibody (Figures 1B,C; Supplementary Figures S1G,H). To assess the endogenous levels of LGG-1 and to avoid potential effects of the GFP fusion, we generated an anti-LGG-1 antibody. We confirmed that the generated antibody allows us to determine LGG-1 protein levels (Supplementary Figures S5A,B). With the LGG-1 antibody we could only detect the total amount of LGG-1 and consequently could not differentiate between the levels of LGG-1-I/II. Nevertheless, we observed that endogenous LGG-1 protein also accumulates in response to knockdown of the chaperone genes thus confirming the reporter analysis (Figures 1D,E).

An accumulation of LGG-1 levels could either stem from an induction of autophagy or result from an impairment of autophagy that would lead to an accumulation of LGG-1. To differentiate between these two possibilities, we analyzed the levels of SQST-1/p62 that acts as an adaptor for the autophagy pathway and more importantly is degraded by autophagy. Thus, the SQST-1/p62 levels can indicate the state of the autophagic flux (Bjorkoy et al., 2005). We generated an SQST-1/p62 antibody and confirmed that the produced antibody recognizes SQST-1/p62 protein (Supplementary Figures S5C,D). As shown in Figures 1D,F,L, the levels of SQST-1/p62 are reduced upon knockdown of the chaperones compared to the control in young adult animals.

The increase of the LGG-1 levels and simultaneous decrease of SQST-1/p62 levels suggest an activation of autophagy in response to a depletion of the chaperones required for disaggregation in *C. elegans*. To eliminate the possibility that our observations are merely due to a general chaperone effect and are not specific to the activity as protein disaggregases, we assessed the LGG-1 and SQST-1/p62 levels upon depletion of chaperones that are not part of the HSP70/110/J-protein families. We choose two small heat shock proteins, *hsp-16.41* and *hsp-17* that are not exhibiting any protein disaggregation activity. Notably, knockdown of *hsp-16.41* or *hsp-17* does not affect the levels of LGG-1 nor SQST-1/p62 (Supplementary Figures S8A,B). We conclude that the induction of autophagy is a specific response to a compromised activity in chaperone-mediated protein disaggregation.

To confirm our observations in mammalian cells we performed siRNA experiments in HEK293 cells and analyzed the autophagic flux. For that we established that the knockdown efficiencies were sufficient using western blot. The remaining protein levels for the three chaperones upon transfection of siRNA were between 40 and 60% (Supplementary Figures S1C,D). Depletion of the orthologous chaperones, *hspa8 (hsp-1), hspa4 (hsp-110) and dnajb1 (dnj-13)*, led to increased levels of LC3-II (Figures 1G,H). The LC3-II levels correlate with the amount of autophagosomes. To differentiate between induction and impairment of autophagic flux, we treated the cells with BafA1, a lysosome V-ATPase inhibitor inhibiting the autophagic flux (Tanida et al., 2005; Fass et al., 2006). In the absence of BafA1, LC3-II levels are elevated upon knockdown of the chaperone genes and the presence of BafA1 caused an increase of LC3-II levels compared to the siRNA control (Figures 1G,H). The elevated LC3-II levels upon BafA1 treatment indicate an induction of autophagy, which is consistent with the data obtained using *C. elegans*.

With the progression of aging misfolded and aggregated proteins are accumulating and pose a burden to the cell (David et al., 2010). These aggregates are potential substrates for either remodeling by the disaggregation complex or clearance by the proteasome or autophagic pathway. The analysis of the autophagy flux during aging has produced contradictory outcomes. While some have reported autophagy activation during aging (Chapin et al., 2015) others have suggested the opposite (Chang et al., 2017). We took advantage of the short lifespan of *C. elegans* to investigate how autophagy is regulated during aging. We observed increased LGG-1 as well as SQST-1/p62 levels from young adults (day 4) to old wt animals (day 10), suggesting an impairment of autophagy flux upon aging (Figures 1I,J) and confirming previous data (Chang et al., 2017).

Based on these data, we analyzed the autophagy flux upon knockdown of the HSP70/110/J complex members at different ages. Confocal microscopy shows an accumulation of GFP::LGG-1 in old (day 10) animals compared to young adults (day 4) for all conditions (Figure 1K). Subsequently, we analyzed the autophagic flux by assessing SQST-1/p62 and LGG-1 protein levels upon chaperone knockdown in more detail in a time-course experiment on day 4, 6, 8 and 10 (Figure 1L). We could observe that at day 6 LGG-1 protein levels peak for all distinct knockdowns and SQST-1/p62 correspondingly exhibits the lowest levels between days 4 and 6. As aging progresses (days 6–10), autophagy appears to be less activated in response to chaperone depletion as we observed diminishing differences in LGG-1 and SQST-1/p62 levels between control and the chaperone knockdowns. These data suggest that a compromised chaperone activity leads to an activation of the autophagy machinery only in young adult animals.

### Chaperone Knockdown Reduces the Lysosome Pool, Leads to an Accumulation of RAB-7 and Initiates Translocation of HLH-30 to the Nucleus

**Figure 2:**
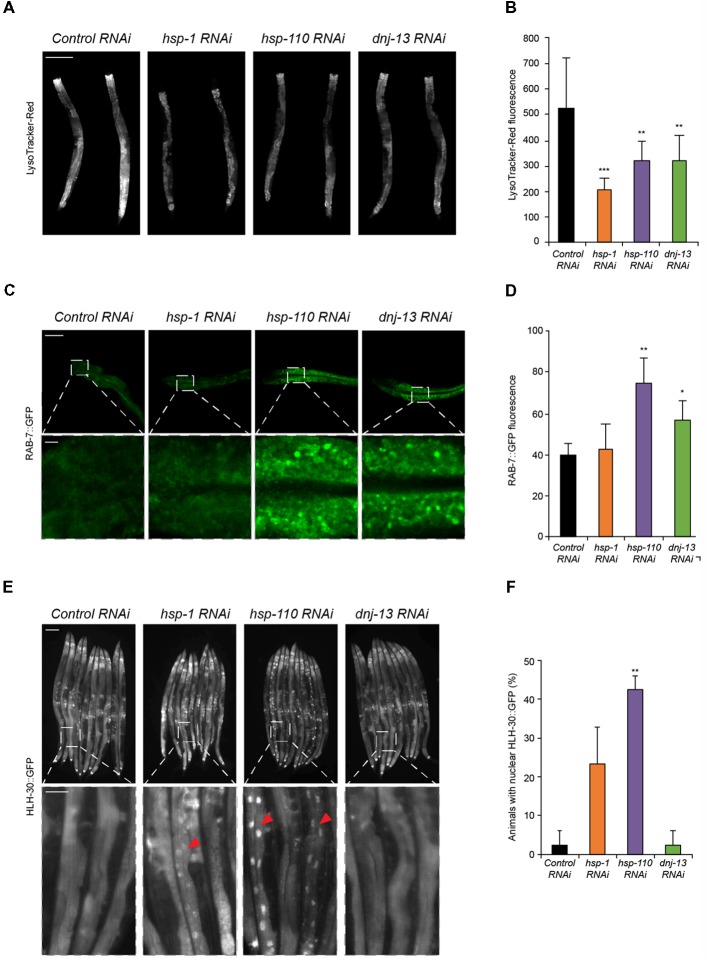
Chaperone knockdown reduces lysosomal pool and initiates HLH-30 translocation to the nucleus. **(A,B)** Lysosomal pool of RNAi treated day 4 old animals. Quantification and normalization of LysoTracker-Red fluorescence **(B)**. The error bars represent the SD of a minimum of 10 animals each. Scale bar in **(A)**: 200 μm. **(C,D)** RAB-7::GFP nematodes were RNAi treated for 4 days. Upper panel shows intestinal cells and the lower panel a magnified region **(C)**. GFP fluorescence was normalized to control RNAi **(D)**. Error bars represent the SD of a minimum of three animals. **(E,F)** HLH-30::GFP animals were RNAi treated for 4 days. Percentage of animals with HLH-30::GFP nuclear translocation is shown **(F)**. Red triangles point out nuclear HLH-30::GFP signals **(E)**. Twenty-five animals per condition were analyzed. Scale bars: 200 μm (upper) and 50 μm (lower panel).

To gain insight into how a depletion of chaperones causes the induction of autophagy we analyzed the lysosome pathway in response to a depletion of the disaggregating chaperones. A crucial step in the autophagy pathway is the fusion between the autophagosomes and lysosomes (Dunn, 1990). Therefore, the lysosomal pool can be used as a read-out for either an impairment (accumulation of lysosomes) or activation of autophagy (decrease of lysosomes). We could detect a notable reduction in the lysosome pool stained with LysoTracker-Red upon knockdown of all three chaperones compared to the control (Figures 2A,B). As controls, we treated the animals with established autophagy modulators and confirmed that activation (rapamycin; rap) led to a reduction of the lysosome pool whereas an impairment (chloroquine; CQ) of autophagy led to an increase of the lysosome pool (Supplementary Figures S2A,B). The observed reduction of the lysosome pool confirms our data of the activation of autophagy upon chaperone depletion, which consumes the lysosome pool (Figure 1).

In addition, we analyzed the vesicular transport that is essential for the autophagy flux. RAB proteins are known to participate in vesicular trafficking. RAB-7 is involved in late-endosomal trafficking and is important for the maturation of autophagosomal structures. Thus, we investigated the effect of chaperone knockdown in a RAB-7::GFP reporter strain (Chen et al., 2006). Depletion of *hsp-110* and *dnj-13*, yet not *hsp-1* led to an accumulation of RAB-7 positive structures in intestinal cells (Figures 2C,D). Consistently, activation of autophagy with rapamycin led to an increase of RAB-7 fluorescence (Supplementary Figures S2C,D).

Transcriptional regulation of autophagy in mammals is controlled by the transcription factor EB (TFEB; HLH-30 in *C. elegans*). To elucidate the mechanism for the activation of autophagy upon depletion of chaperones, we used an HLH-30::GFP reporter strain (Lapierre et al., 2013). Notably, depletion of *hsp-1*, but particularly of *hsp-110* led to a nuclear translocation of HLH-30 (Figures 2E,F). We conclude that the activation of autophagy upon chaperone knockdown is regulated *via* HLH-30, which translocates to the nucleus upon autophagy induction as shown by rapamycin treatment (Figures 2E,F; Supplementary Figure S2E). Taken together, these results confirm that the autophagy-lysosome pathway is activated when the chaperone complex is compromised.

### Proteasome Capacity Is Diminished When HSP-110/70/J Machinery Is Compromised

**Figure 3:**
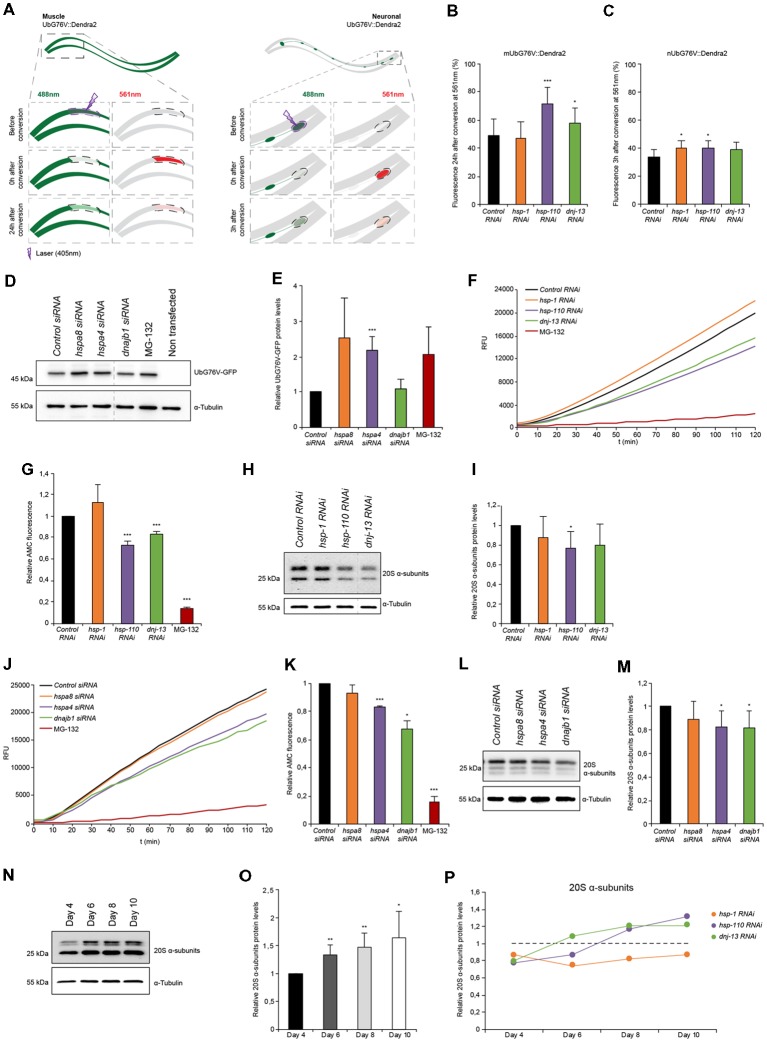
Proteasome capacity is diminished when HSP-110/70/J machinery is perturbed. **(A)** Scheme of the single-cell photo-conversion of UbG76V::Dendra2 in muscle and neuronal cells of *C. elegans*. **(B,C)** RNAi treated UbG76V::Dendra2 animals of muscle **(B)** or neuronal cells **(C)**. UbG76V::Dendra2 protein was analyzed 3 (neurons) or 24 (muscle) hours post conversion at 561 nm. **(B)** Error bars represent the SD of a minimum of nine animals. **(D,E)** Immunoblotting of siRNA transfected HEK293 cells, expressing UbG76V-GFP. Cells treated with 20 μM MG-132 for 4 h served as control. The relative UbG76V-GFP levels are shown **(E)**. Error bars represent the SD of three independent experiments. **(F,G)** Chymotrypsin-like proteasome activity of RNAi treated nematodes for 4 days. Shown are the relative activities **(G)**. Error bars represent the SD of three independent experiments. **(H,I)** Relative protein levels of 20S α-subunits RNAi treated animals. **(I)** Error bars represent the SD of three independent experiments. **(J,K)** Chymotrypsin-like proteasome activity of siRNA treated HEK293 cells. The relative activities are depicted **(K)**. The error bars represent the SD of three independent experiments. **(L,M)** Relative protein levels of 20S α-subunits of siRNA HEK293 cells. **(M)** Error bars represent the SD of a minimum of six independent experiments. **(N,O)** 20S α-subunit levels were determined of nematodes of 4, 6, 8 and 10 days of age and normalized to day 4 old animals **(O)**. Error bars represent the SD of five independent experiments. **(P)** 20S α-subunits at day 4, 6, 8 and 10 of RNAi treated animals. Depicted are the averages of the relative 20S α-subunit levels from a minimum of three independent experiments.

Misfolded and aggregated proteins are also substrates for proteolytic turnover by the UPS. Analogous to our analysis of the crosstalk between autophagy and chaperone-mediated disaggregation we were wondering how the UPS responds to a depletion of disaggregating chaperones. As an *in vivo* read-out for UPS activity, we used UbG76V::Dendra2 that is expressed either in the body wall muscle tissue or in neuronal cells of *C. elegans* (Figure 3A; Supplementary Figure S3A; Hamer et al., 2010). Dendra2 can be irreversibly photo-converted (488 nm (green) to 561 nm (red)) upon excitation at 405 nm. Dendra2 is fused to a non-cleavable ubiquitin moiety (UbG76V) allowing the correct poly-ubiquitination and subsequent targeting of the Dendra2 fusion protein for proteasomal degradation. As reported previously, UbG76V::Dendra2 accumulates when proteasome function is compromised such as upon RNAi of *pas-5* (Supplementary Figure S3B; Hamer et al., 2010). This sensor enables the quantification of proteasome activity in the neuronal and muscle tissue of a whole animal or on a single-cell level by monitoring the red fluorescence post photo-conversion.

In the muscle cells we observed a pronounced accumulation of photo-converted UbG76V::Dendra2 (+45%) when *hsp-110* was depleted compared to the control 24 h after photo-conversion (Figure 3B). This data indicates a reduction of proteasome activity in muscle cells upon *hsp-110* depletion. A decrease in proteasome activity could also be observed upon *dnj-13* knockdown (Figure 3B).

The turnover rates of UbG76V::Dendra2 were much faster in neuronal cells compared to muscle tissue and we therefore measured the stability 3 h post photo-conversion. Depletion of *hsp-1* (+18%) and *hsp-110* (+16%) led to a slight accumulation of UbG76V::Dendra2 compared to the control in neurons (Figure 3C).

To validate our observations in mammalian cell culture, we performed a similar experiment in HEK293 cells where we transfected the non-cleavable ubiquitin moiety fused to GFP (UbG76V-GFP; Dantuma et al., 2000). We could confirm that siRNA of *hspa4 (hsp-110*) led to a significant accumulation (2-fold) of UbG76V-GFP (Figures 3D,E). Moreover, we could also observe reduced degradation upon depletion of *hspa8* (*hsp-1*), suggesting that depletion of disaggregating chaperones exerts an inhibitory effect on UPS activity in mammalian cells, too.

The data obtained with the UbG76V::Dendra2 sensor reflects the *in vivo* proteasomal activity in single muscle and neuronal cells in response to a systemic depletion of the individual chaperone genes. To expand the analysis of the proteasome activity to the whole organism, we measured the *ex vivo* proteasome activity in protein lysates of either HEK293 cells or complete nematodes using a fluorogenic proteasome-specific substrate (AMC) to assess the chymotrypsin-like proteasome activity (Kisselev and Goldberg, 2005). As depicted in Figures 3F,G, animals depleted for *hsp-110* and *dnj-13* display a reduction of 20%–30% of AMC release over time compared to the control indicating reduced proteasome activity. This observation corroborates the *in vivo* data, especially for *hsp-110* RNAi (Figures 3B,C). To distinguish between reduced activity and lower abundance of the proteasome upon knockdown of the chaperones, we quantified the relative protein levels of the 20S α-subunits. Interestingly, only the depletion of *hsp-110* led to significant lower levels of 20S α-subunits compared to the control (Figures 3H,I). Also, we detected reduced mRNA levels of three proteasome components, *rpn-6.1* (19S), *pas-5* (20S α-subunit) and *pbs-5* (20S β-subunit) upon *hsp-1* and *hsp-110* knockdown (Supplementary Figure S4D). These observations suggest that the reduced UPS capacity upon chaperone depletion is likely due to a reduction in the actual amount of the proteasome subunits. As controls we depleted again *hsp-16.41* and *hsp-17* that are not involved in protein disaggregation and we did not observe a reduction of the 20S α-subunit levels and consequently also no inhibitory effect on the chymotrypsin-like proteasomal activity (Supplementary Figures S8C,D).

In HEK293 cells, a diminished chymotrypsin-like proteasome activity could also be observed upon *hspa4* and *dnajb1* siRNA compared to control condition (Figures 3J,K). Analogous to the data obtained for the nematodes, the 20S α-subunits are also reduced in response to *hspa4* and *dnajb1* depletion in the mammalian cells (Figures 3L,M).

Next, we set out to analyze the activity and levels of the proteasome with the progression of aging to complement the data set obtained for the autophagic flux (Figure 1I). For that, we determined the proteasome activity *in* and *ex* *vivo* in older animals. We detected reduced levels of the photo-converted UbG76V::Dendra2 in muscle cells on day 7 (32%) compared to day 4 (49%), yet increased UbG76V::Dendra2 levels in neurons on day 7 (48%) compared to day 4 (34%; Supplementary Figures S4A,B). These data suggest a moderate increase of proteasome activity with the progression of aging in muscle cells, yet a reduction of UPS activity in neurons as previously reported (Hamer et al., 2010). Interestingly, we could detect elevated 20S α-subunit levels of whole nematode lysates with the progression of aging (Figures 3N,O). Next, we measured the 20S α-subunit protein levels upon chaperone depletion with the progression of aging (Figure 3P). 20S α-subunits are more abundant upon knockdown of *hsp-110* and *dnj-13*, yet not of* hsp-1* in older animals (days 8 and 10) compared to the control. However, even though *hsp-110* and *dnj-13* depleted animals display higher 20S a-subunit levels on day 8 compared to the control (Figure 3P), their UPS activity is not increased in old animals compared to the control (Supplementary Figures S4A–C).

These results indicate that depletion of chaperones either in *C. elegans* or HEK293 cells are associated with reduced 20S levels and consequently diminished proteasome function in young animals and cells. In older animals however, the UPS activity is not affected by the depletion of chaperones despite elevated 20S levels.

### Depletion of Chaperones Leads to Protein Aggregation, Induction of Autophagy and Compromised UPS Activity

**Figure 4:**
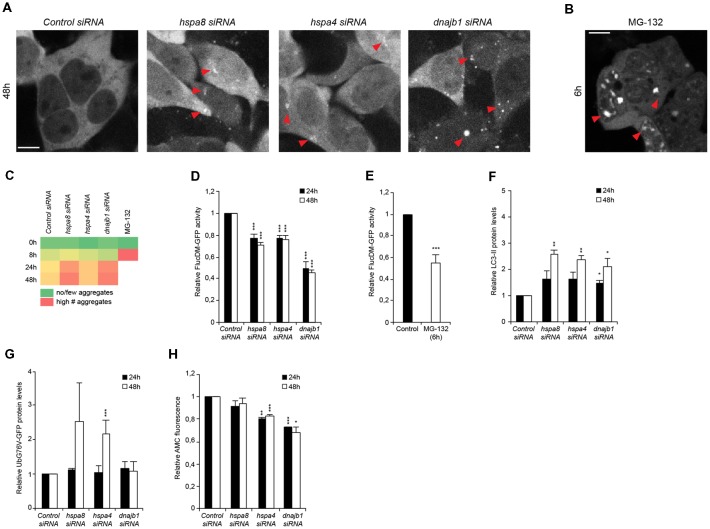
Depletion of chaperones promotes cytosolic stress, protein misfolding and is associated with an induction of autophagy and diminished ubiquitin proteasome system (UPS) capacity. **(A,B)** siRNA treated HEK293 cells expressing FlucDM-GFP were subjected to live-cell imaging. Depicted are representative images 48 h post siRNA transfection **(A)**. As control, cells were treated with 20 μM MG-132 for 6 h **(B)**. Red triangles point out FlucDM-GFP aggregates. Scale bars: 10 μm. **(C)** Aggregation analysis of the images acquired at 0, 8, 24 and 48 h of **(A,B)**. The green color represents no or a low number of cells with aggregates, an intermediate number of cells with aggregates is indicated in yellow and the red color indicates a high number of cells with aggregates. **(D,E)** FlucDM-GFP activity 24 and 48 h after siRNA transfection. Relative luciferase activity of the chaperone knockdown conditions and the respective time point **(A)**. As control, cells were treated with 20 μM MG-132 for 6 h **(E)**. Error bars represent the SD of three independent experiments. **(F)** LC3-II levels of siRNA treated HEK293 cells. Depicted are normalized LC3-II levels. **(G)** UbG76V-GFP levels of siRNA treated HEK293 cells. **(H)** Chymotrypsin-like proteasome activity of siRNA treated HEK293 cells.

To better understand the order of events upon and how loss of HSP70/110/J affects the cellular folding capacity and the crosstalk with the proteolytic pathways, we designed a time-course siRNA experiment in HEK293 cells. We employed a well-established protein-folding sensor, a GFP-tagged destabilized luciferase variant, FlucDM-GFP, (Gupta et al., 2011) that misfolds and aggregates rapidly in response to proteostasis perturbations. Using this sensor, we aimed to determine the time-point upon chaperone depletion when luciferase starts to aggregate and loses its enzymatic function to correlate it to the induction of autophagy and inhibition of UPS capacity. We performed a live-cell imaging experiment where the chaperones were depleted, and the cells imaged for 48 h. The formation of the first foci representing aggregated FlucDM-GFP in response to chaperone depletion was detected 24 h post siRNA transfection. At 48 h of siRNA treatment, the chaperone knockdown conditions displayed visible accumulation of aggregated luciferase particularly upon depletion of *hspa8* and *dnajb1* (Figures 4A–C).

In parallel, we monitored the enzymatic activity of the luciferase sensor in intact cells in a luminescence-based assay. By measuring the luciferase activity at different time points after chaperone depletion, we were able to confirm that the loss of luciferase activity occurred already 24 h after siRNA transfection for all chaperone siRNA conditions and most prominently for *dnajb1* (Figure 4D). MG-132 was used as a control (Gupta et al., 2011), and we could observe major luciferase aggregation 6 h after compound treatment, which corresponded to a decrease of 45% of luciferase activity (Figures 4B,E).

These data led us to inquire if the protein clearance mechanisms were already affected when the cells were treated with siRNA for 24 h. We could observe that LC3-II levels were already slightly increased at 24 h after transfection and further increased to the 48-h siRNA time-point (Figure 4F). An accumulation of the UPS substrate UbG76V-GFP could only be detected at the 48-h post siRNA treatment time point for depletion of *hspa4* (Figure 4G). Yet, a reduction of chymotrypsin-like proteasome activity is detected already at the 24 h time-point in *hspa4* and *dnajb1* siRNA conditions (Figure 4H). This suggest that the UPS activity is already compromised 24 h post transfection, yet an accumulation of UPS substrates becomes prominent only 48 h post siRNA-mediated depletion of the chaperones.

In sum, these results suggest that depletion of chaperones leads to an accumulation of protein aggregates, which triggers the induction of autophagy and simultaneously leads to a reduction of proteasome capacity.

### Autophagy Is Impaired in Neurodegenerative-Disease *C. elegans* Models

**Figure 5:**
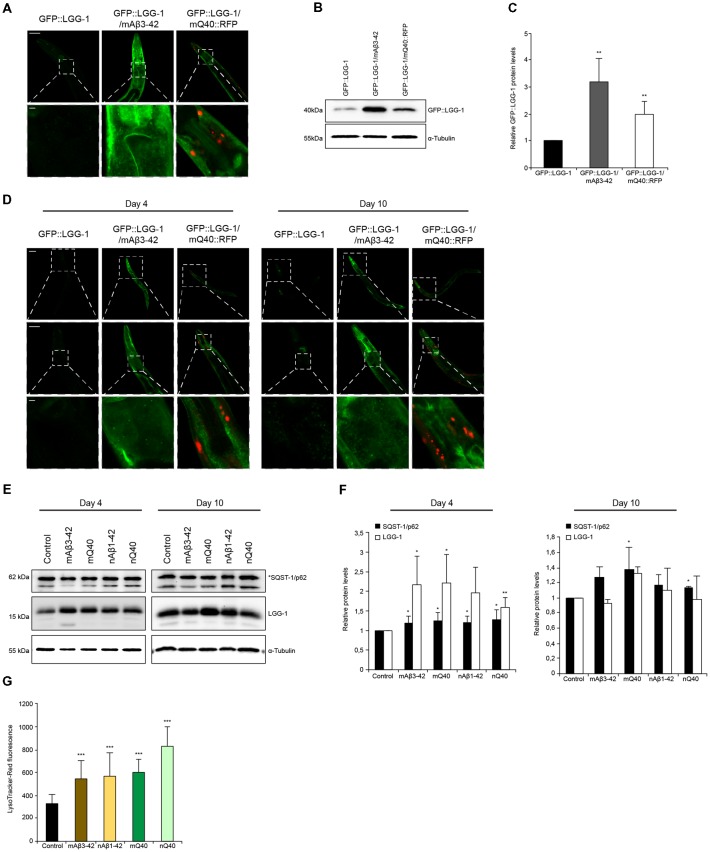
Autophagy is impaired in neurodegenerative-disease *C. elegans* models. **(A)** GFP::LGG-1, GFP::LGG-1; mAβ_3–42_ and GFP::LGG-1; mQ_40_::RFP animals at day 5. The lower panel is a magnification of the head region. Scale bars: 50 μm (upper) and 5 μm (lower panel). **(B,C)** GFP::LGG-1 levels of control, Aβ_3–42_ and Q_40_ animals at day 5. Relative intensities of GFP::LGG-1 are shown **(C)**. Error bars represent the standard deviation from four independent experiments. **(D)** GFP::LGG-1, GFP::LGG-1; mAβ_3–42_ and GFP::LGG-1; mQ_40_::RFP animals at day 4 (left panel) and day 10 (right panel). Upper, middle and lower panels show whole animal, head and a magnification of the head region, respectively. Scale bars: 20 μm (upper), 50 μm (middle) and 5 μm (lower panel). **(E,F)** LGG-1 and SQST-1/p62 levels of control, mAβ_3–42_, nAβ_3–42_, mQ_40_::RFP and nQ_40_::YFP day 4 and 10 old nematodes. Normalized LGG-1 and SQST-1/p62 levels. Error bars represent the SD of a minimum of three independent experiments. **(G)** LysoTracker staining of control, mAβ_3–42_, nAβ_1–42_, mQ_40_::RFP and nQ_40_::YFP day 4 animals. The error bars represent the SD of a minimum of 12 animals for each condition.

Aggregation and accumulation of toxic protein aggregates are a hallmark of neurodegenerative disorders (Ross and Poirier, 2004). It is known that the presence of such toxic proteins destabilizes cellular and organismal proteostasis. We set out to analyze how the protein degradation machineries are affected by two distinct and well-established *C. elegans* models that express amyloid-like proteins, Aβ_3–42_ and polyglutamine repeats. We crossed animals expressing Aβ_3–42_ or polyglutamine (Q_40_) in the body wall muscle tissue with animals expressing the autophagy reporter GFP::LGG-1. The expression of Aβ_3–42_ and Q_40_ led to an overall increase of GFP::LGG-1 fluorescence (Supplementary Figure S6C), but the muscle tissue and head region showed the most prominent GFP::LGG-1 accumulation (Figure 5A). The accumulation of GFP::LGG-1 protein in Aβ_3–42_ (3-fold) and Q_40_ (2-fold) expressing animals relative to the control was also confirmed by western blot (Figures 5B,C).

In addition, we analyzed the levels of SQST-1/p62::GFP in animals expressing Aβ_3–42_ in the muscle (Supplementary Figure S6D). The absence of fluorescence tagging of Aβ_3–42_ enables a reliable quantification of SQST-1/p62::GFP puncta. The Aβ_3–42_ expressing animals display a significant increase of SQST-1/p62::GFP puncta, although the puncta are smaller compared to the control (Supplementary Figures S6D,E). Also, it is detected an accumulation of SQST-1/p62::GFP puncta in the muscle tissue, contrary to the control which comprise the majority of SQST-1/p62::GFP puncta in close proximity to the pharynx.

Neurodegenerative diseases are generally late age of onset diseases (Morimoto and Cuervo, 2014). To understand how the presence of disease-associated protein aggregates affects the autophagy pathway, we analyzed the autophagic flux on day 4 (young) and on day 10 (old) in Aβ_3–42_/Q_40_ animals. We could observe that both young and old Aβ_3–42_/Q_40_ expressing nematodes display increased GFP::LGG-1 fluorescence compared to control animals (Figure 5D). As depicted in Figures 1I–K, wt animals show elevated LGG-1 levels with the progression of aging. However, in Aβ_3–42_/Q_40_ animals the GFP::LGG-1 signal at day 10 was similar to day 4 (Figure 5D). This observation led us to determine the relative protein levels of LGG-1 and SQST-1/p62 in young and old animals expressing the disease proteins. We analyzed animals expressing Aβ_3–42_ and Q_40_ in the body-wall muscle and in neuronal cells. All disease proteins lead to a strong increase of LGG-1, but also a moderate increase of SQST-1/p62 in young adults, indicating an impairment of autophagy (Figures 5E,F). As aging progresses, the impact of the disease proteins becomes less pronounced and the LGG-1 and SQST-1/p62 levels are not or only slightly elevated compared to control animals. This is supported by the fact that old animals exhibit an already impaired autophagic system (Figures 1I,J). The observation of an accumulation of the lysosome pool confirms our data of a compromised autophagic flux in Aβ_3–42_ and Q_40_-expressing animals (Figures 5G).

### Amyloid Protein Aggregation Leads to Proteasome Impairment Across Tissues

**Figure 6:**
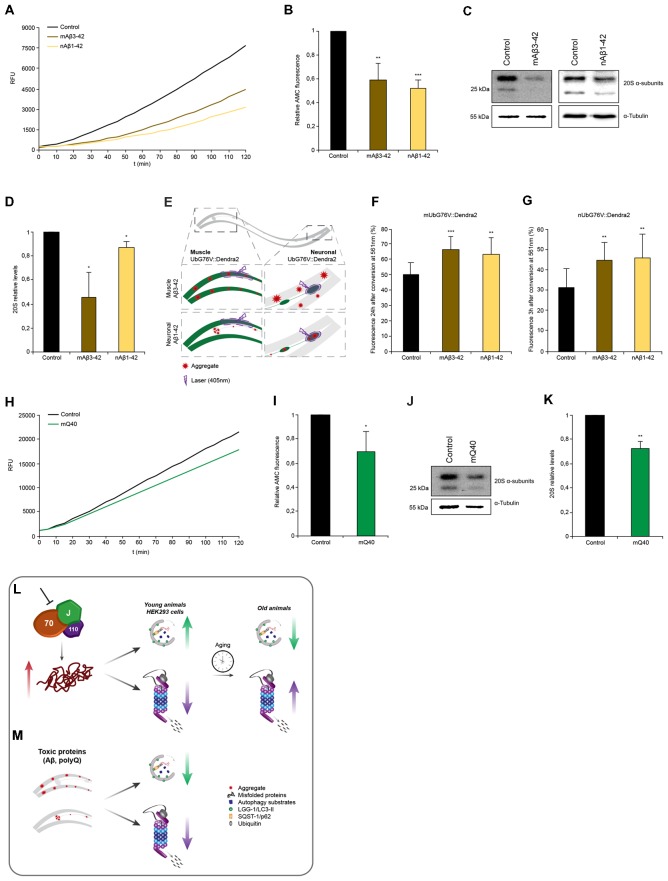
Proteotoxic stress caused by amyloidogenic aggregates is associated with proteasome impairment. **(A,B)** Chymotrypsin-like proteasome activity of control, nAβ_1–42_ and mAβ_3–42_ day 4 animals. Depicted are relative activities **(B)**. Error bars represent the standard deviation of three independent experiments. **(C,D)** 20S α-subunit levels of control, nAβ_1–42_ and mAβ_3–42_ day 4 animals. Depicted are relative 20S levels **(D)**. Error bars represent the SD of three independent experiments. **(E)** Representative scheme of the UbG76V::Dendra2 and Aβ_3–42_ crossed animals. Degradation of UbG76V::Dendra2 in the muscle cells was determined in the presence of Aβ aggregates in muscle and neurons (left) and the degradation rates of UbG76V::Dendra2 in the neuronal cells were measured in the presence of Aβ aggregates in muscle and neurons (right). Aggregates are indicated as red stared shapes. **(F,G)** UbG76V::Dendra2 degradation rates in the indicated disease models. Converted UbG76V::Dendra2 protein was imaged immediately and 3 **(G)** or 24 **(F)** h later as in Figures 3B,C. Error bars represent the SD of a minimum of 11 animals. **(H,I)** Chymotrypsin-like proteasome activity of control and mQ_40_::YFP day 4 animals. Depicted are normalized activities **(I)**. Error bars represent the SD of three independent experiments. **(J,K)** 20S α-subunit levels of control and mQ_40_::YFP day 4 animals. Depicted are normalized 20S levels **(K)**. Error bars represent the SD of three independent experiments. **(L,M)** Model: depletion of HSP70/110/J disaggregating chaperones leads to an increase of misfolded proteins, which is associated with an induction of autophagy and an impairment of proteasome function in young animals and HEK293 cells **(L)**. With the progression of aging in *C. elegans*, autophagy is not activated and UPS activity is no longer reduced in response to chaperone loss. Autophagy and proteasome pathways are perturbed in the presence of amyloid-like protein aggregates in the muscle and neurons of *C. elegans* **(M)**.

Next, we wanted to assess the proteasome capacity of animals expressing the disease proteins. We observed that nematodes expressing Aβ_3–42_ either in muscle cells (−40%) or in neuronal cells (−48%) exhibited reduced chymotrypsin-like proteasome activity compared to control animals (Figures 6A,B). The 20S α-subunits are also reduced in these animals and that likely cause or at least contribute to the reduction in activity similarly to our observations upon chaperone depletion (Figures 6C,D). As the chymotrypsin-like proteasome activity reflects the activity on an organismal level, we decided to analyze the proteasome activity in distinct tissues. For that we crossed animals expressing Aβ_3–42_ with the *in vivo* neuronal and muscle UPS sensor UbG76V::Dendra2 (Figure 6E). Both, the muscle and neuronal model, express Aβ_3–42_ without a fluorescence tag enabling an accurate Dendra2 quantification. Animals expressing Aβ_3–42_ in the muscle tissue displayed reduced degradation rates in muscle (−30%) and neurons (−45%; Figures 6F,G). Analogously, Aβ_3–42_ expressed in neurons also led to a diminished proteasome activity in muscle (−25%) and neuronal cells (−48%). These data show that neurodegenerative disease associated proteins inhibit proteasome function within the same and notably also across tissues.

Interestingly, whereas expression of Q_40_ in muscle tissue led to a reduction of chymotrypsin-like proteasome activity of 30% and analogously of the 20S α-subunit levels (Figures 6H–K), neuronal expression of Q_40_ did not affect proteasomal activity (Supplementary Figure S6A). Neither animals expressing a longer polyQ stretch (Q_67_) in the neurons, show any proteasome activity decline (Supplementary Figure S6B).

In addition, we demonstrated that the expression of the aggregation-prone HttExon1Q_97_-GFP in HEK293 cells also leads to a reduction chymotrypsin-like proteasome activity by 16% (Supplementary Figures S7A–D) as previously shown for similar Htt aggregation-prone constructs (Bence et al., 2001). The soluble HttExon1Q_25_-GFP protein did not affect the chymotrypsin-like proteasome activity and served as control (Supplementary Figures S7A–D). Unlike the *C. elegans* data, the expression of mHttExon1Q_97_-GFP in HEK293 cells did not alter the 20S levels (Supplementary Figures S7E,F).

## Discussion

In this study, we analyzed the crosstalk between the disaggregase constituting chaperones and the two major proteolytic pathways, the UPS and autophagy in response to the expression of disease-associated amyloid proteins and with the progression of aging.

Aging is marked by an accumulation of misfolded and aggregated proteins. It has been established that the heat shock response is repressed with the onset of reproduction. The inducibility of chaperone gene expression in response to proteotoxic challenges declines sharply once adulthood is reached and further declines with the progression of aging (Ben-Zvi et al., 2009). Our understanding of the regulation of the proteolytic pathways during aging is far less understood and not consensual. Previous analyses focused on either the UPS or autophagy and employed different model systems and consequently applied different experimental conditions. In this manuscript we used with *C. elegans* a well-established aging model system and analyzed both, the autophagic flux and the UPS capacity, in parallel using the same growth and experimental conditions and thus minimizing experimental deviations. Here, we could show that autophagy is less active with the progression of aging. We also observed that autophagy is induced when members of the chaperone disaggregation complex are depleted in *C. elegans* and HEK293 cells. Yet, the induction of autophagy is diminished in old animals. Thus, the compensatory effect of autophagy to maintain proteostasis is limited to the reproductive period of animals.

Notably, autophagy is only activated in response to the depletion of disaggregating chaperones and not chaperones that are not involved in the resolubilization of protein aggregates. Most chaperones fulfill pleiotropic roles and are involved in a number of different protein folding, sorting or remodeling activities. It is thus challenging to single out distinct chaperones of the Hsp70/110/J-protein families that only exhibit disaggregation activity. This is in particular challenging for the Hsp70s that are involved in numerous protein folding events, but also in the targeting of substrates to the proteasome or chaperone-mediated autophagy (Esser et al., 2004; Kriegenburg et al., 2012; Cuervo and Wong, 2014). This also explains why the phenotype of *hsp-1*/*hspa8* depletions in the autophagy and UPS assays were not as strong as for instance for the knockdown of *hsp-110*. The only biological role described so far for HSP-110 is the nucleotide exchange factor function as part of protein disaggregation (Rampelt et al., 2012; Nillegoda et al., 2015; Kirstein et al., 2017). Moreover, the genome of *C. elegans* encodes for only one *hsp-110* gene. As such it is not that surprising that *hsp-110* depletion shows the strongest phenotype on autophagy and UPS capacity of the tested chaperones as the disaggregase activity is completely abrogated upon knockdown of *hsp-110* (Rampelt et al., 2012; Nillegoda et al., 2015; Kirstein et al., 2017).

Contrary to autophagy, the proteasome capacity is compromised upon knockdown of specifically the disaggregating chaperones. We detected a decrease in the proteasome 20S α-subunit levels upon chaperone depletion. Thus, the reduced UPS activity is likely due to either a decreased synthesis or stability of the 20S subunits. The observed induction of autophagy upon depletion of chaperones suggests the latter and might be responsible for a potential degradation of proteasome subunits, which has not yet been described in *C. elegans* (Marshall et al., 2015). Another possible scenario could be a defective proteasome complex assembly and subsequent instability of the individual subunits (Hammack et al., 2017). Interestingly, the 20S α-subunit levels increase with the progression of aging on an organismal level. Yet the comparison of different tissues revealed a cell-type specific proteasome regulation. While old animals display a moderately increased proteasome activity in muscle cells, the UPS activity declines in neurons (Figures 6L,M).

How could a depletion of the disaggregating chaperones lead to an activation of autophagy? It is possible that the specific chaperones of the HSP70/110/J-protein complex directly affect the signaling pathways activating autophagy by e.g., chaperoning components of the autophagy pathway. Yet it is also likely that autophagy is activated more indirectly by an accumulation of protein aggregates in the absence of those chaperones that clear aggregated proteins. The latter scenario is supported by our observation of an absence of any compensatory mechanism of the UPS in response to reduced disaggregation capacity. In fact, depletion of disaggregating chaperones even leads to a reduction of 20S subunits and correspondingly diminished UPS capacity. Thus, a depletion of disaggregating chaperones compromises protein aggregate resolubilization as well as proteolytic clearance by the UPS that consequently leads to an accumulation of aggregating proteins (Figure 4).

Neurodegenerative diseases are characterized by a terminal accumulation of proteotoxic protein aggregates excluding a recovery due to failure of the protein aggregate clearance pathways. We observed that the expression of disease-associated amyloid proteins causes an impairment of autophagy irrespective of the type of amyloid (Aβ_3–42_ and Q_40_) and expression pattern (neuronal and muscle tissue). And while it has been demonstrated that proteasomes can degrade aggregation-prone proteins, various studies show that amyloid proteins can lead to an impairment of proteasome function (Bence et al., 2001; Lopez Salon et al., 2003; Juenemann et al., 2013; Thibaudeau et al., 2018). Here, we also demonstrate that the presence of Aβ_3–42_ and Q_40_ diminishes the proteasome capacity in *C. elegans* as well as in HEK293 cells. We could further demonstrate for the first time that the Aβ_3–42_ protein aggregates can also suppress the UPS activity in a distal tissue. This suggests either a trans-tissue communication to adjust the UPS capacity in response to perturbations of proteostasis or that Aβ_3–42_ can spread between tissues and thereby exert its negative effect on the UPS activity. Indeed, spreading of Aβ_3–42_ between neighboring cells has been recently demonstrated in *C. elegans* (Melentijevic et al., 2017).

## Author Contributions

DF performed all nematode and most cell culture experiments. KJ aided DF with some cell culture experiments and helped in the set-up of some assays. MI generated the *hsp-17* and *hsp-16.41* RNAi clones and the HSP-17 antibody. IB assisted DF with some proteasome assays. CH contributed reagents and technology transfer. JK supervised this project. DF and JK wrote the manuscript with input from all authors.

## Conflict of Interest Statement

The authors declare that the research was conducted in the absence of any commercial or financial relationships that could be construed as a potential conflict of interest.
