# Supplementary material for: Crosstalk Between Chaperone-Mediated Protein Disaggregation and Proteolytic Pathways in Aging and Disease
Source: Front Aging Neurosci. 2019 Jan 29;11:9. doi: 10.3389/fnagi.2019.00009 (PMC6361847; doi:10.3389/fnagi.2019.00009)
Supplement: Supplementary file 1 [file Table_1.docx]

**Supporting information**

**
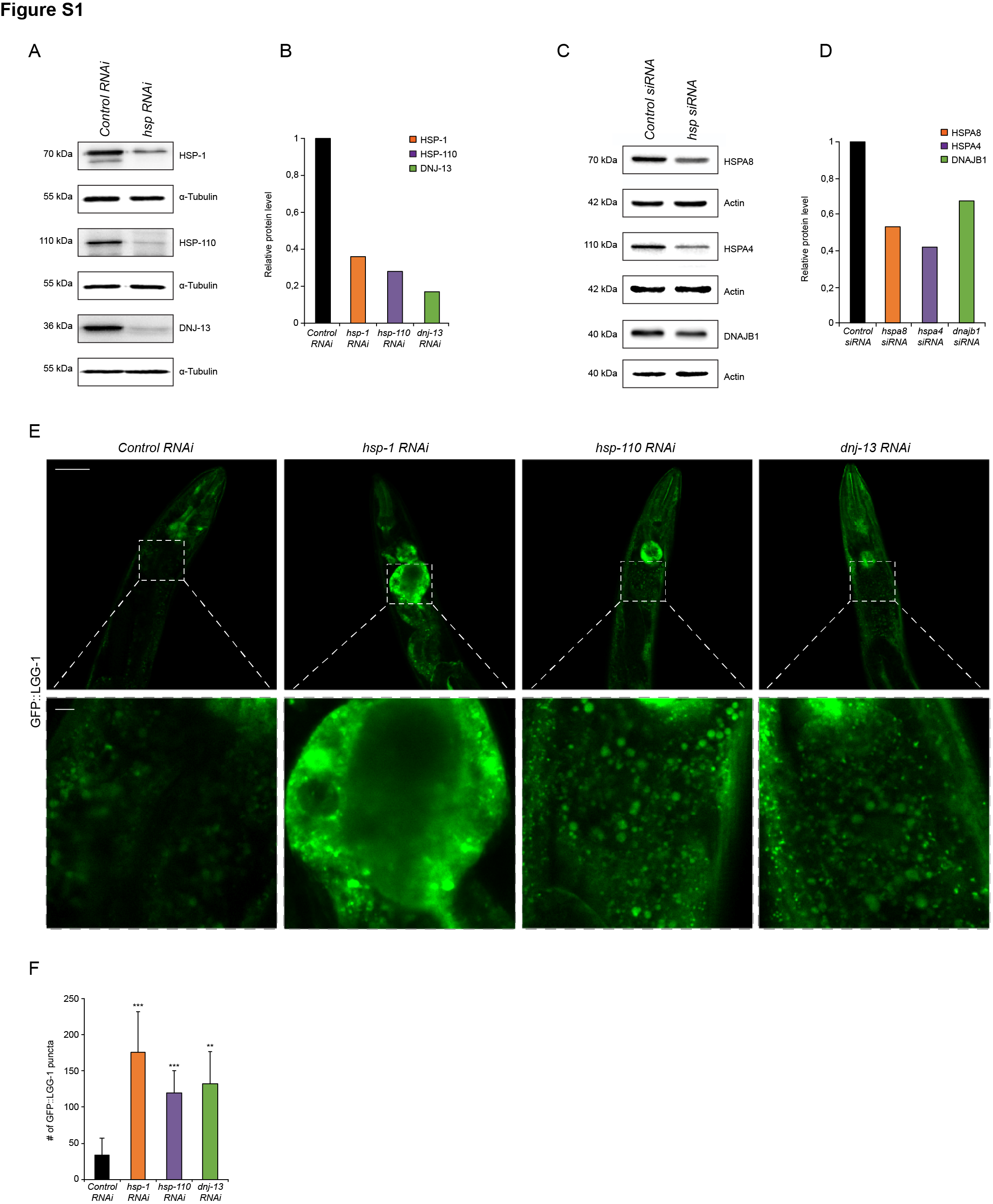
**

**Figure S1. Analysis of RNAi and siRNA chaperone knockdown efficiencies in *C. elegans* and HEK293 cells, and GFP::LGG-1 expression pattern upon chaperone depletion and disease models**

A-B) Western blot of chaperone levels of day 4 nematodes treated with RNAi against the indicated chaperones. The relative band intensity of each chaperone was normalized to α-tubulin level and then to control RNAi (B). C-D) Chaperone levels of HEK293 cell lysates which were transfected with the indicated siRNA for 48 hours. The relative band intensity of each chaperone was normalized to actin levels and then to control siRNA (D). E) Fluorescent images of GFP::LGG-1 animals (day 6) subjected to the indicated RNAi (as shown in Figure 1A). Scale bars: 50 μm (upper) and 5 μm (lower panel). F) Quantification of GFP::LGG-1 puncta in the intestine region posterior to the pharynx (area depicted in the lower panel of E)) of animals (day 6) subjected to the indicated RNAi. The error bars represent the standard deviation of a minimum of 4 animals. G+H) Immunoblotting of GFP::LGG-1 RNAi treated animals (as shown in Figure 1B). GFP cleaved product levels were normalized to α-tubulin levels and then to RNAi control (H). The dashed lines indicate that some lanes were removed from the immunoblot.

**
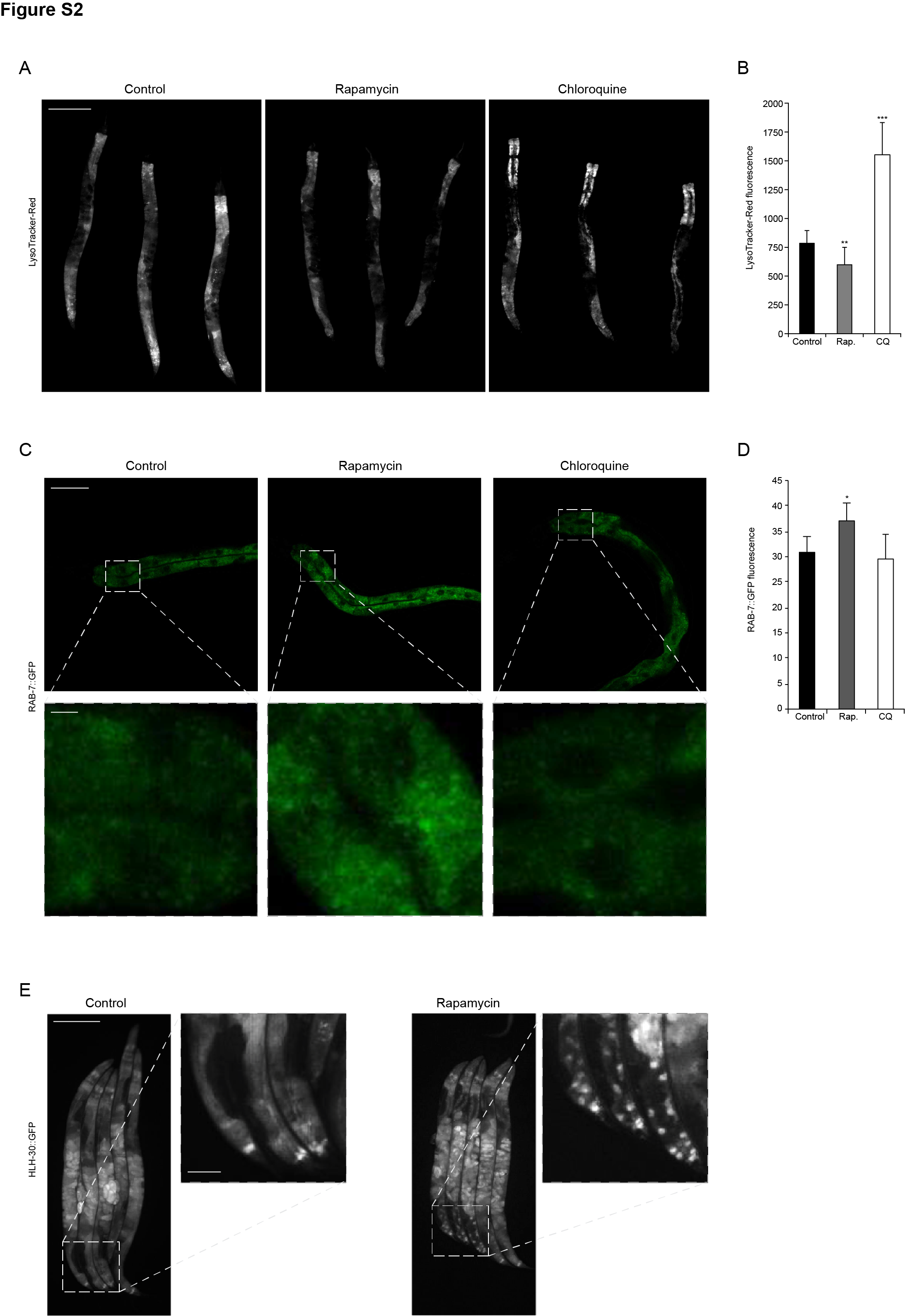
**

**Figure S2. Modulation of the autophagy pathway**

A-B) Lysosomal pool of day 4 animals treated with 200 μM Rapamycin or 10 mM chloroquine for 20 hours was stained with LysoTracker-Red. LysoTracker-Red fluorescence was quantified and normalized to control animals. The error bars represent the standard deviation of a minimum of 4 animals. Scale bar: 200 μm. C-D) Day 4 RAB-7::GFP expressing nematodes were imaged after 20 hours of 200 μM Rapamycin or 10 mM chloroquine treatment. The upper panel shows the intestinal cells of the nematodes and the lower panel depicts a magnified region of the upper panel (C). GFP fluorescence was quantified and normalized to control animals (D). The error bars represent the standard deviation of a minimum of 5 animals. Scale bars: 10 μm. E) Day 4 animals expressing HLH-30::GFP were treated with 200 μM Rapamycin for 20 hours or left untreated. The HLH-30::GFP nuclear translocation was subsequently analyzed in 10 animals. Scale bars: 200 μm (left panel) and 50 μm (right panel, magnification).

**
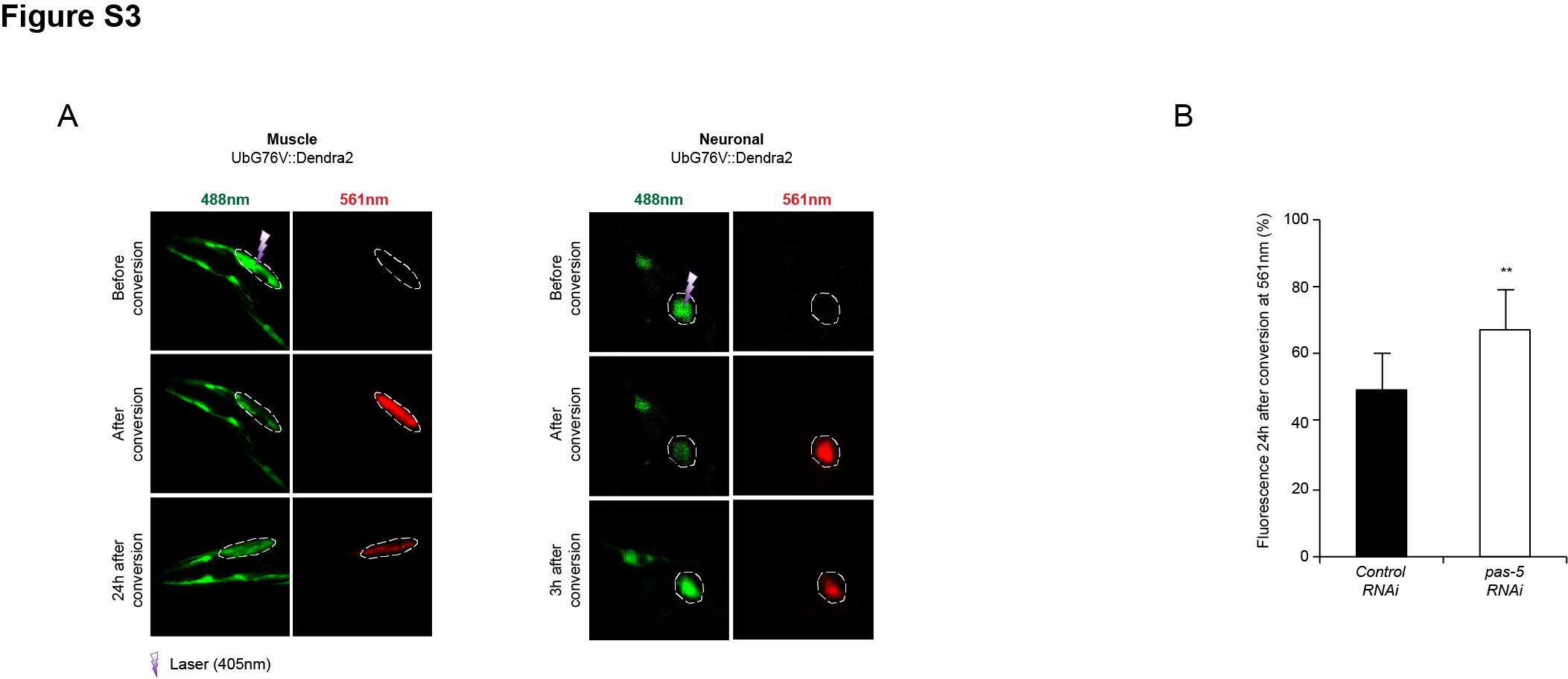
**

**Figure S3. PAS-5 knockdown reduces the degradation rates of UbG76V::Dendra2**

A) Photo-conversion of UbG76V::Dendra2 in muscle (left side) and neuronal (right side) cells of *C. elegans*. The dashed line encircles the photo-converted region upon exposure to 405 nm. The photo-converted UbG76V::Dendra2 protein was analyzed 3 (neurons) or 24 (muscle) hours post conversion at 561 nm.

B) Animals expressing mUbG76V::Dendra2 were treated with *pas-5* RNAi for 72 hours. UbG76V::Dendra2 protein was converted and the animals were imaged 24 hours post conversion as in figure 3B. The error bars represent the standard deviation of a minimum of 5 animals.

**
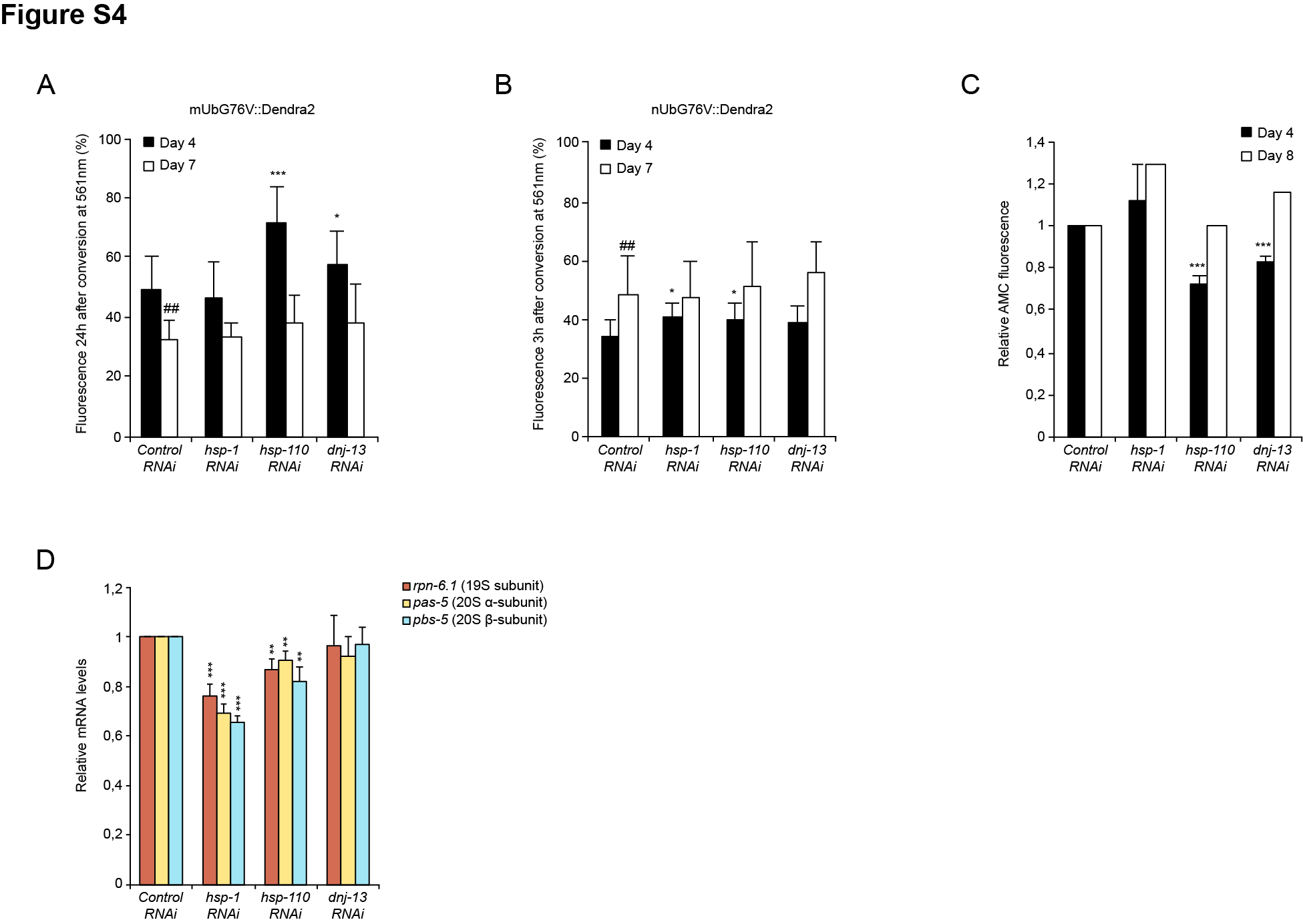
**

**Figure S4. Proteasome activity is altered with the progression of aging, and the mRNA of proteasomal genes is affected by chaperone depletion**

A-B) The degradation rates of UbG76V::Dendra2 animals were measured at day 4 and day 7 of age in muscle (A) or neuronal cells (B). UbG76V::Dendra2 protein was converted and the animals were imaged 3 or 24 hours after conversion (as in Figs. 3B-C). The error bars represent the standard deviation of a minimum of 6 animals. The significance between day 4 and day 7 of the control is indicated by the character # (p<0,01, ##). C) Chymotrypsin-like proteasome activity of wt nematodes treated with the indicated RNAi for 4 or 8 days. The relative activities were calculated and normalized to RNAi control. Error bars represent the standard deviation of 3/1 (day 4/ day 8) experiments. D) Relative mRNA levels of *rpn-6.1*, *pas-5* and *pbs-5* in animals (day 4) treated with the indicated RNAi. Error bars represent the standard deviation of three independent experiments.

**
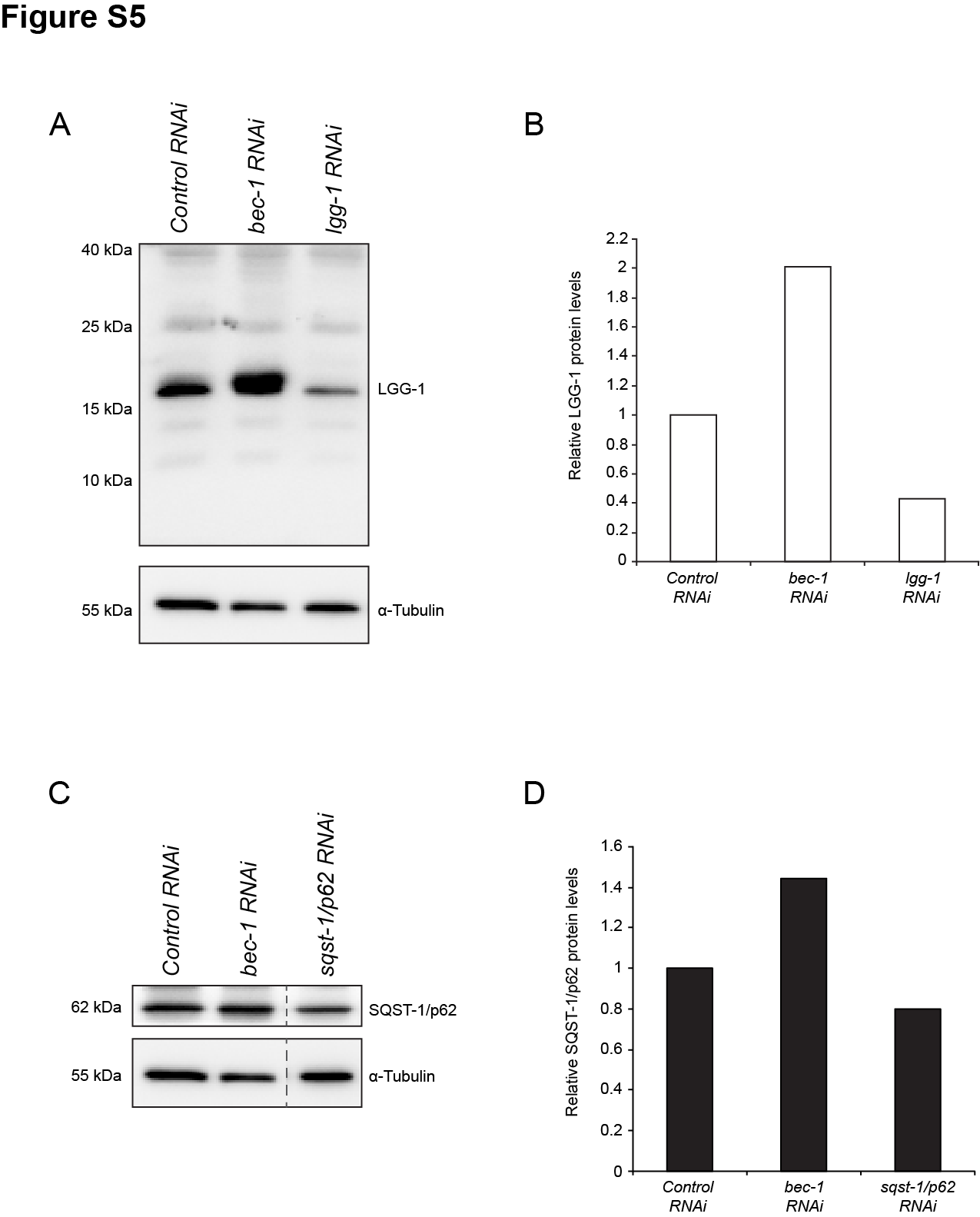
**

**Figure S5. LGG-1 and SQST-1/p62 levels upon genetic modulations**

A-B) LGG-1 levels from day 4 wt animals treated the indicated RNAi. The LGG-1 levels were normalized to α-tubulin levels and then to RNAi control (B). C-D) SQST-1/p62 levels from day 4 wt animals treated the indicated RNAi. SQST-1/p62 levels were normalized to α-tubulin levels and then to RNAi control (D).

**­­­**

**
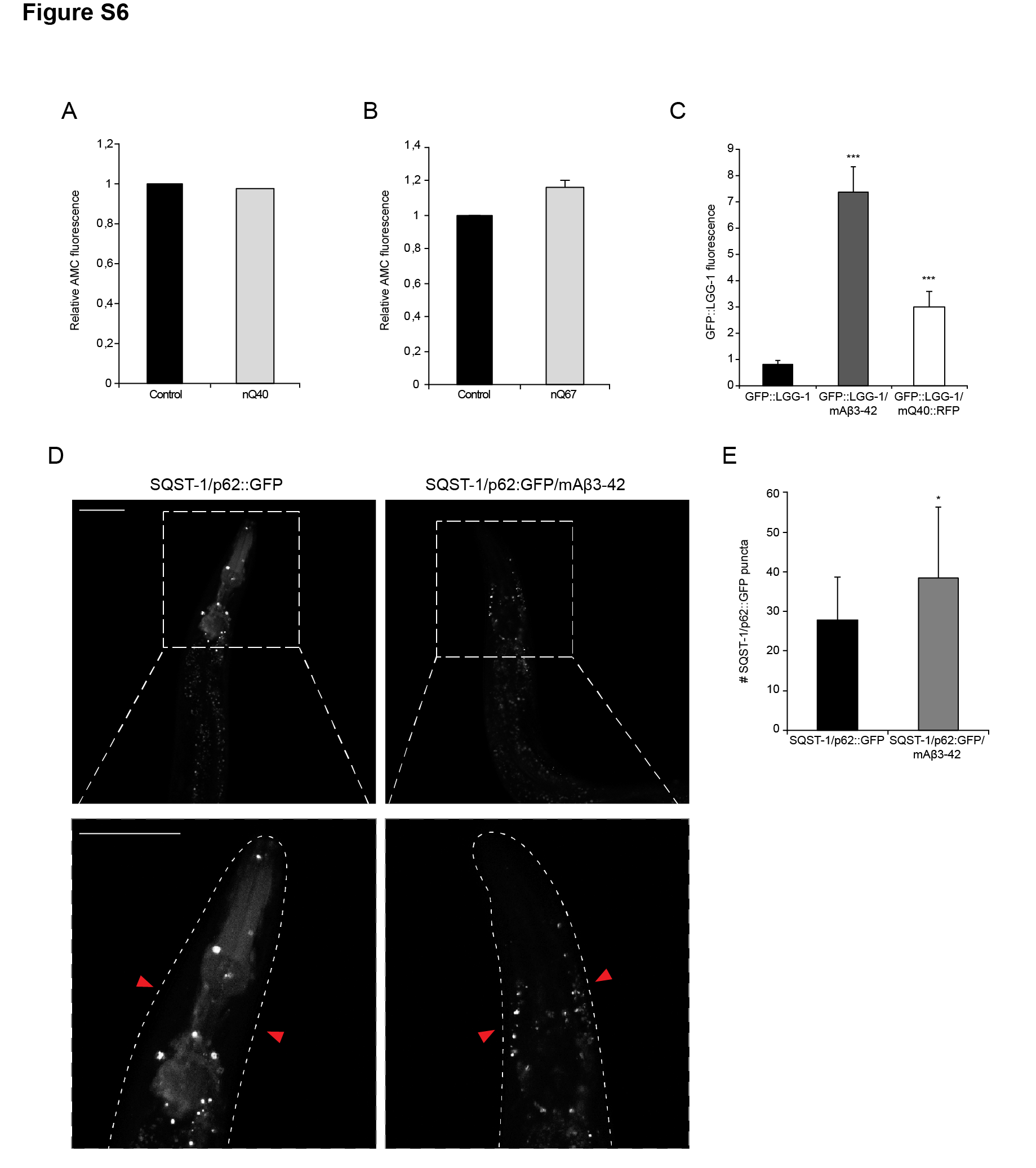
**

**Figure S6. Analysis of proteasome and autophagy systems in disease models**

A) Chymotrypsin-like proteasome activity of control and nQ_40_::YFP day 4 animals. The relative activities were calculated and normalized to control. B) Chymotrypsin-like proteasome activity of control and nQ_67_::CFP day 4 animals. The relative activities were calculated and normalized to control. The error bars represent the standard deviation of two independent experiments. C) Quantification of GFP::LGG-1 fluorescence of GFP::LGG-1, GFP::LGG-1; mAβ_3-42_ and GFP::LGG-1; mQ_40_::RFP animals at day 5. The error bars represent the standard deviation of a minimum of 6 animals. D) Fluorescent images of SQST-1/p62::GFP and SQST-1/p62::GFP; mAβ_3-42_ animals at day 5. In the lower panel the animals are lined out by the dashed lines, and the red triangles are pointing to the muscle tissue. Scale bars: 50 μm E) Quantification of SQST-1/p62::GFP puncta in the head region (D). The error bars represent the standard deviation of a minimum of 19 animals.

**
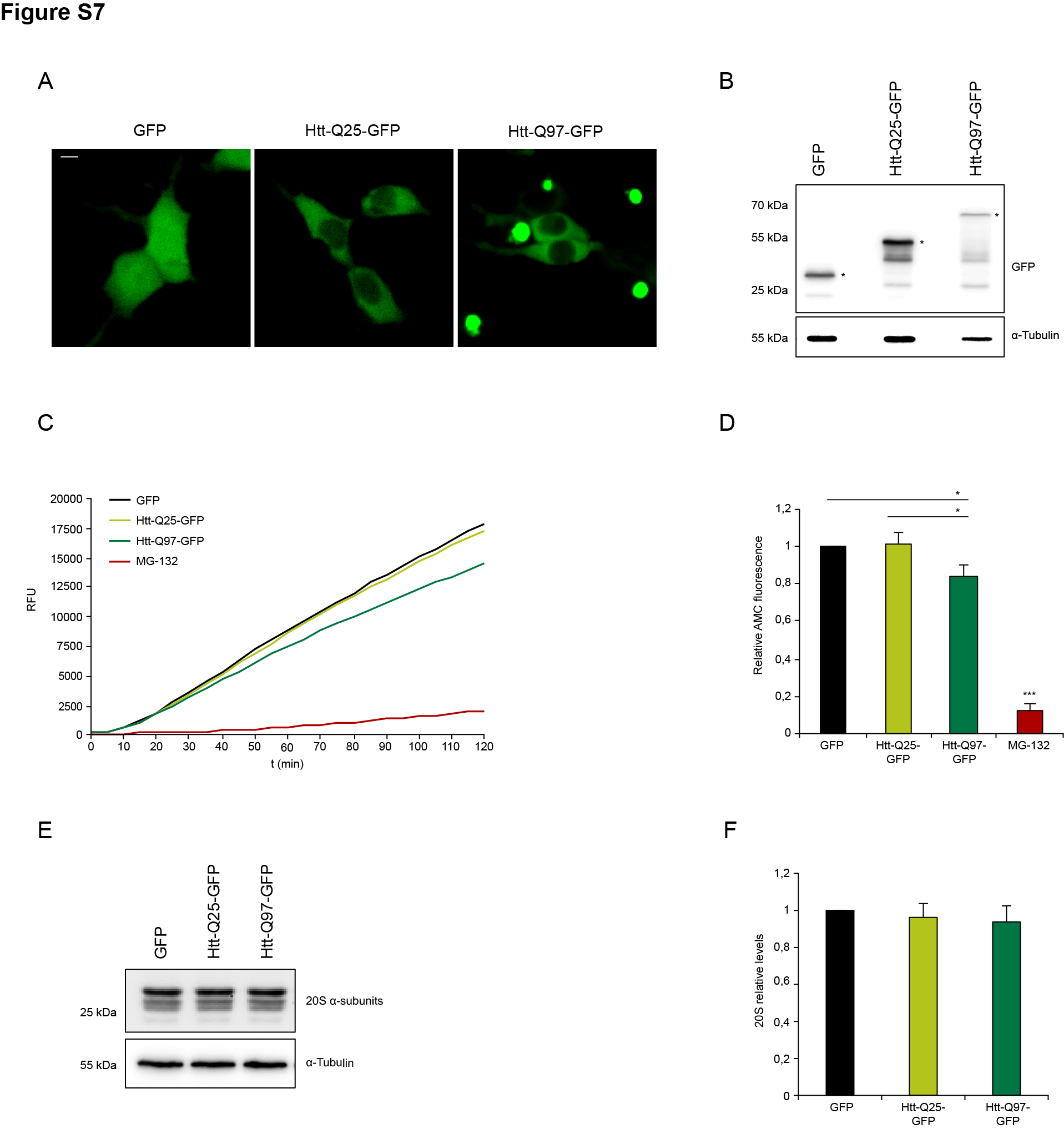
**

**Figure S7. Expression of HttExon1Q_97_ aggregates are associated with reduced proteasome activity in HEK293 cells**

A-B) HEK293 cells were transfected with GFP, HttExon1Q_25_-GFP and HttExon1Q_97_-GFP and imaged 24 hours post transfection (A). The expression of GFP, HttExon1Q_25_-GFP and HttExon1Q_97_-GFP was also confirmed by western blot (B). Scale bar: 5 μM. C-D) Chymotrypsin-like proteasome activity of HEK293 cells transfected with GFP, HttExon1Q_25_-GFP or HttExon1Q_97_-GFP. The relative activities were calculated and normalized to GFP. Treatment with MG-132 served as control (D). The error bars represent the standard deviation of three independent experiments. E-F) 20S α-subunit levels were determined in HEK293 cells transfected with GFP, HttExon1Q_25_-GFP or HttExon1Q_97_-GFP. The 20S levels were normalized to α-tubulin levels and then to GFP (F). The error bars represent the standard deviation of four independent experiments.

**
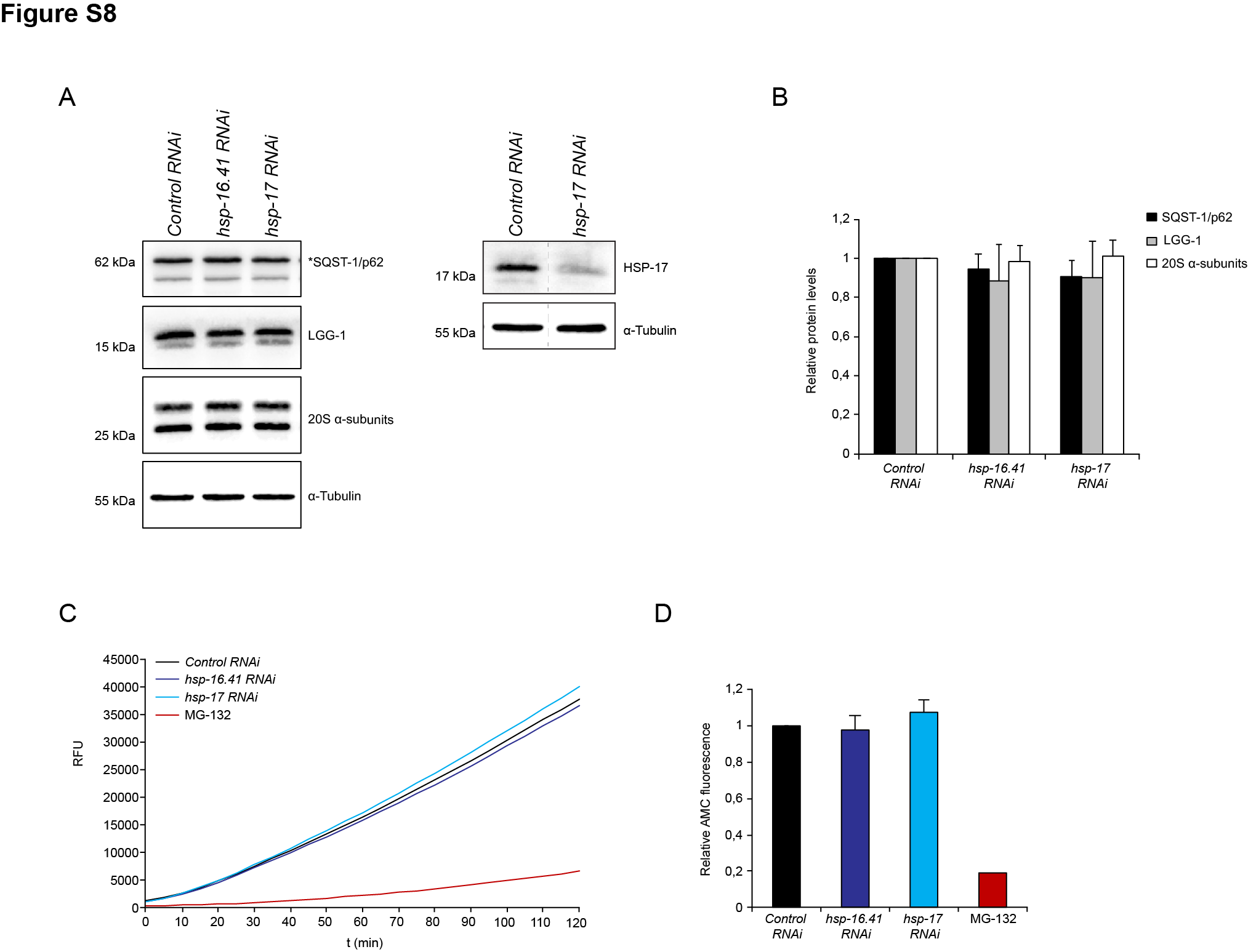
**

**Figure S8. *hsp-16.41* and *hsp-17* depletion does not affect autophagy and proteasome pathways**

A-B) LGG-1, SQST-1/p62 and 20S levels of nematodes treated with the indicated RNAi for 4 days. LGG-1, SQST-1/p62 and 20S levels were normalized to RNAi control (B). The error bars represent the standard deviation from five independent experiments.

C-D) Chymotrypsin-like proteasome activity of nematodes treated with the indicated RNAi for 4 days. The relative activities were calculated and normalized to RNAi control (D). The error bars represent the standard deviation of two independent experiments.
