# Supplementary figures and images for: Crosstalk Between Chaperone-Mediated Protein Disaggregation and Proteolytic Pathways in Aging and Disease
Source: Front Aging Neurosci. 2019 Jan 29;11:9. doi: 10.3389/fnagi.2019.00009 (PMC6361847; doi:10.3389/fnagi.2019.00009)

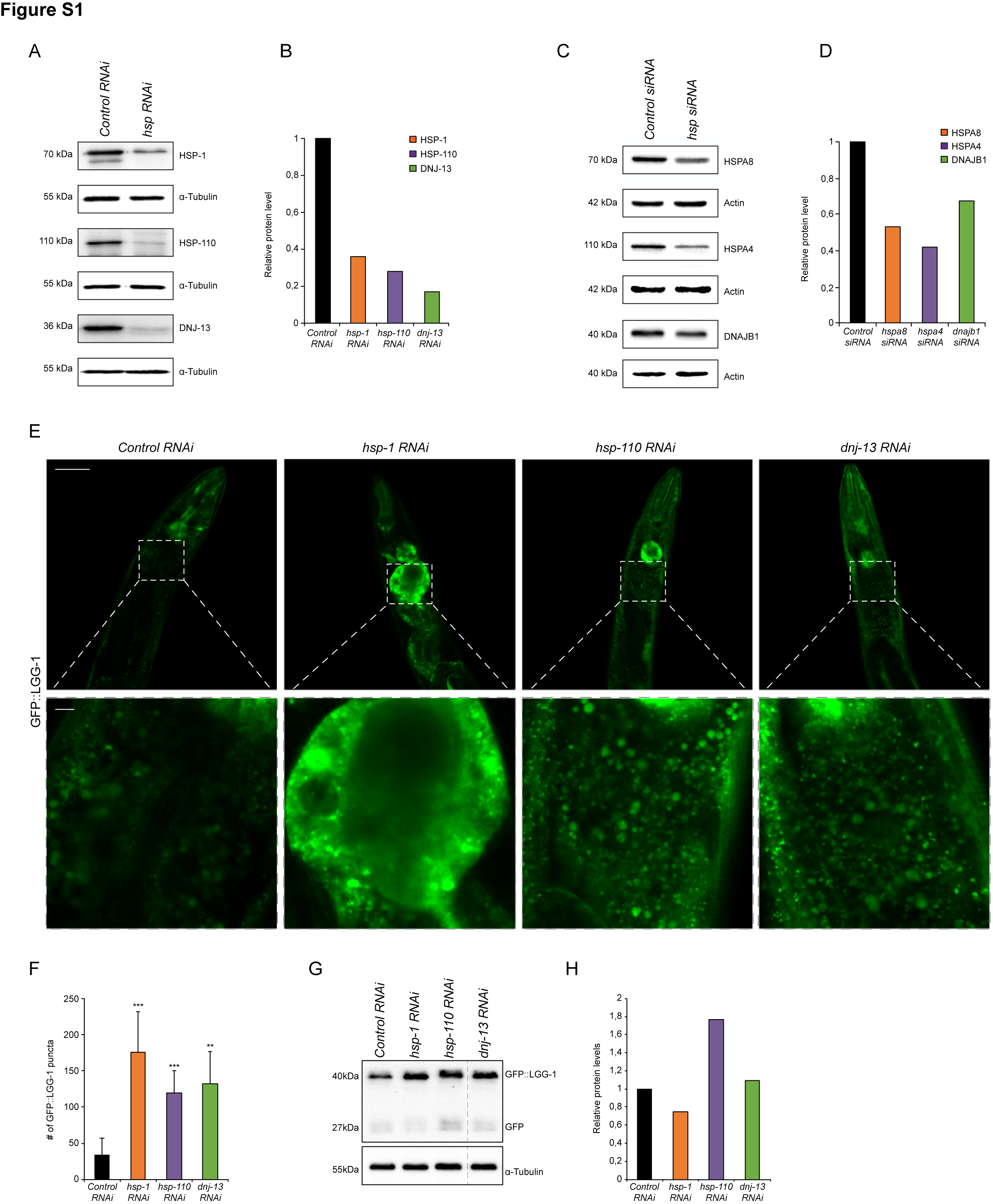

Supplement: Supplementary file 2 [file Image_1.TIF]

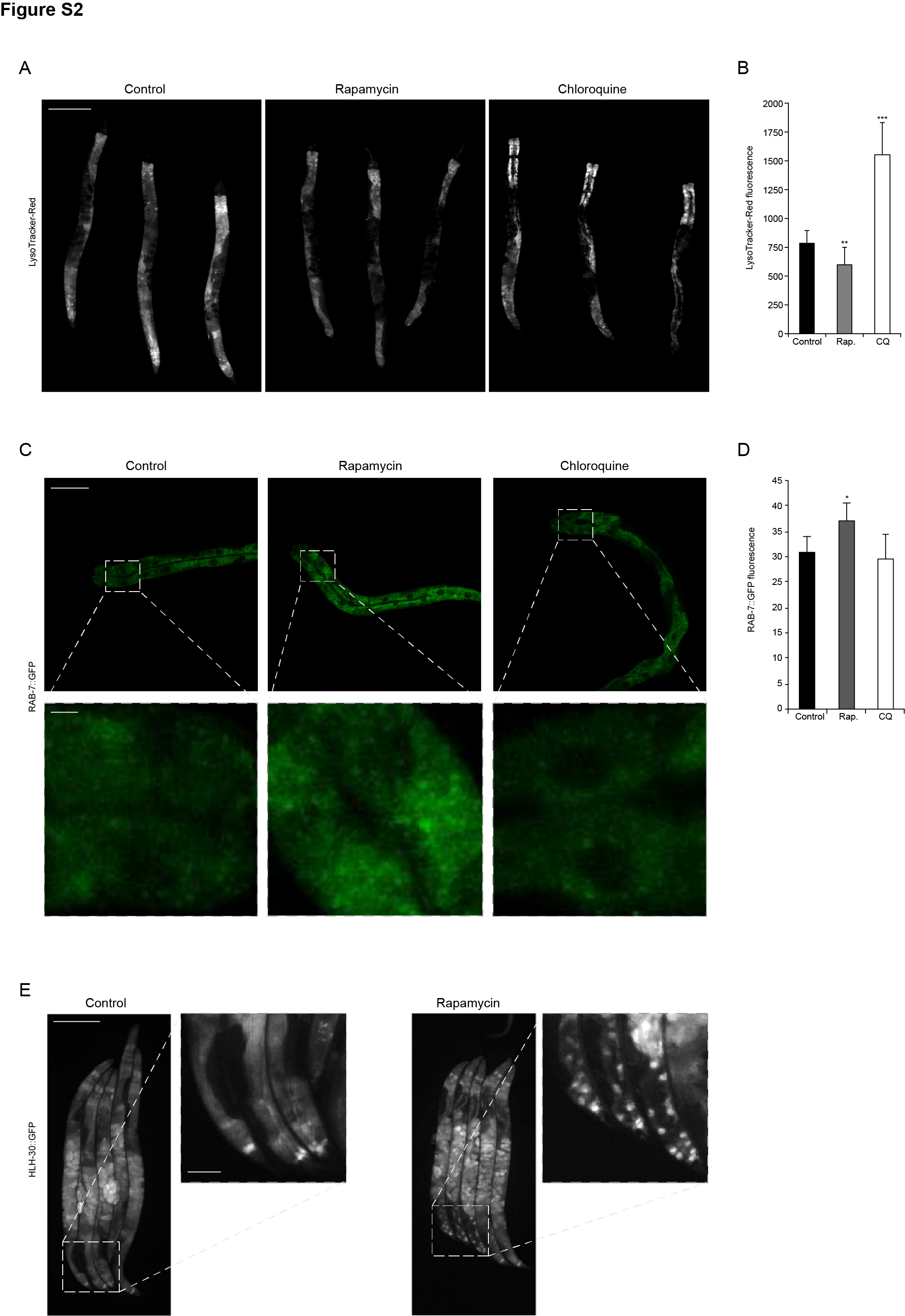

Supplement: Supplementary file 3 [file Image_2.TIF]

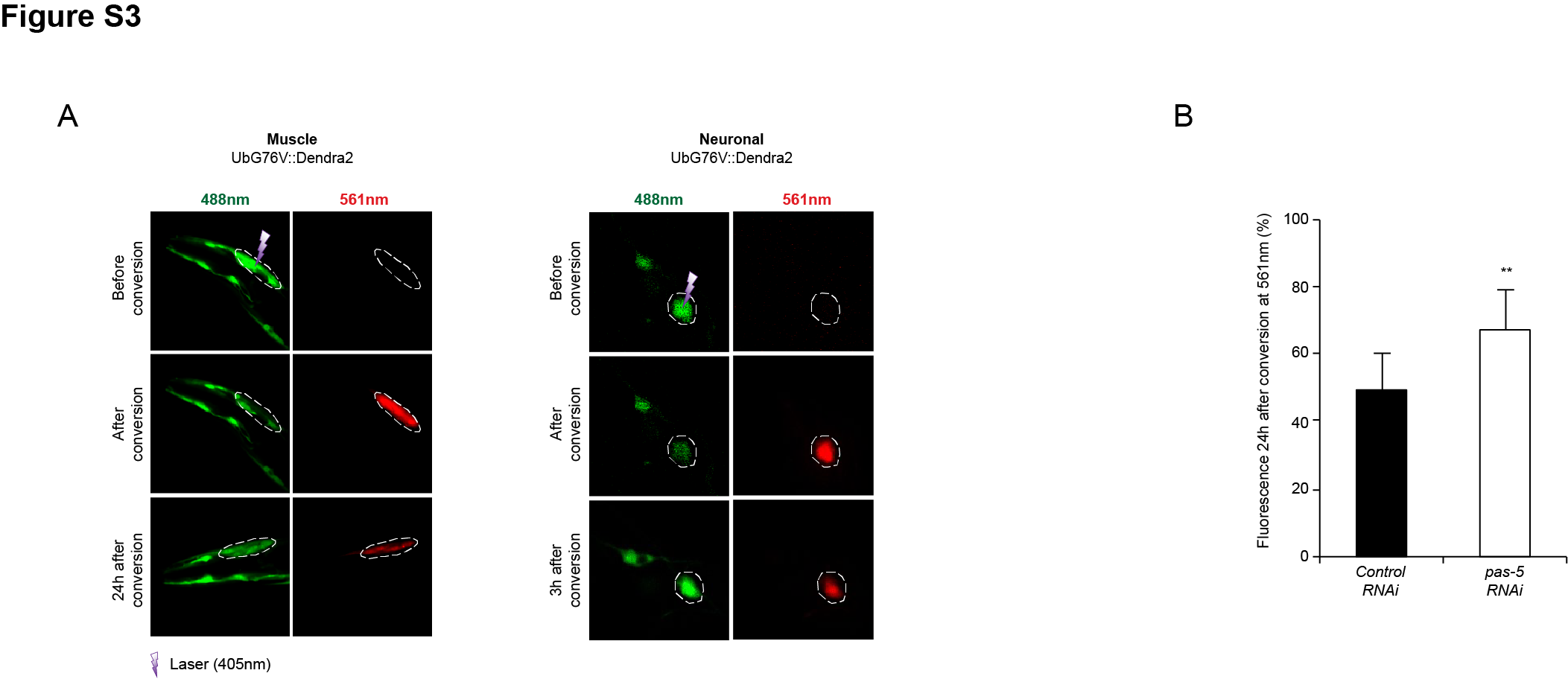

Supplement: Supplementary file 4 [file Image_3.TIF]

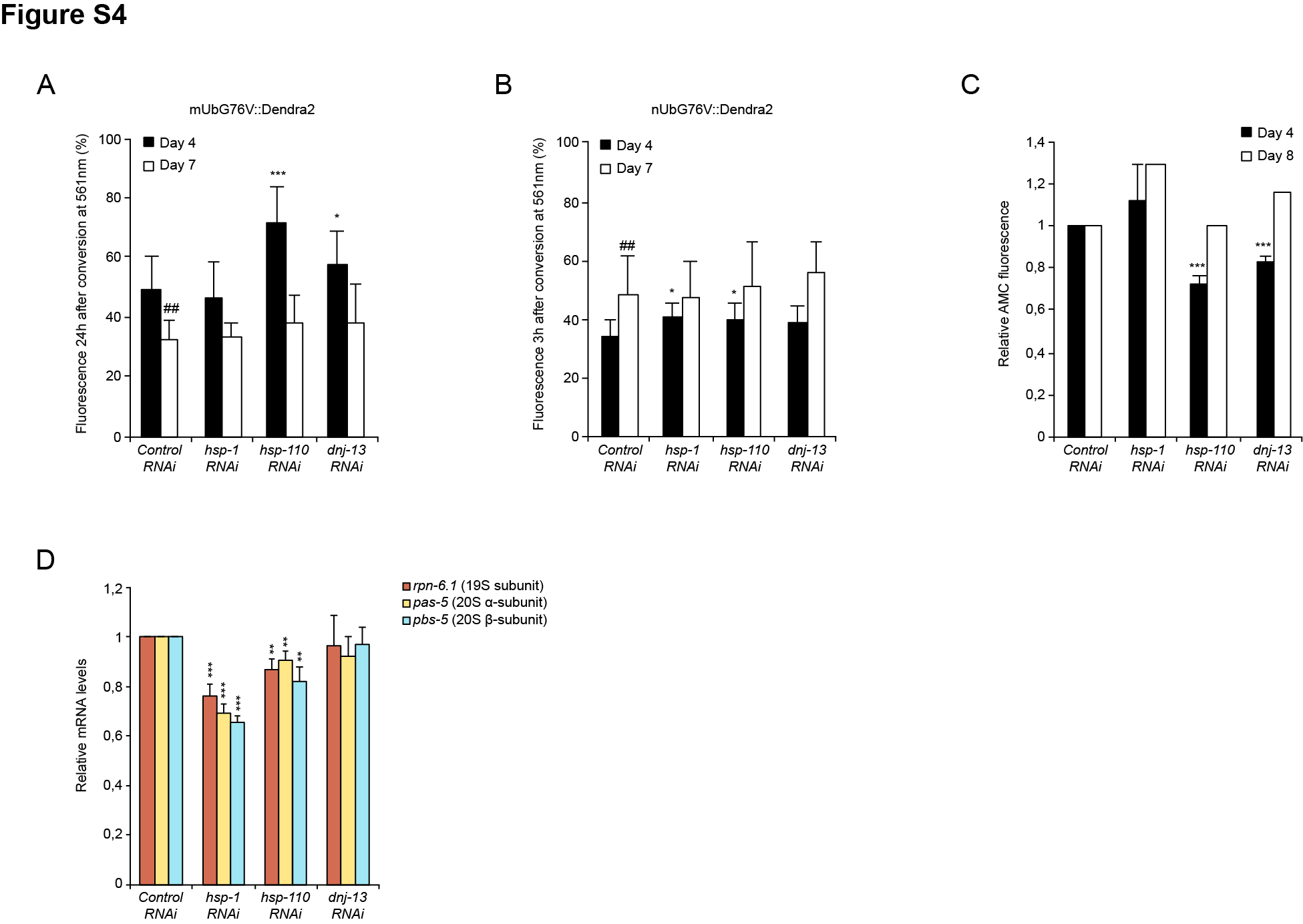

Supplement: Supplementary file 5 [file Image_4.TIF]

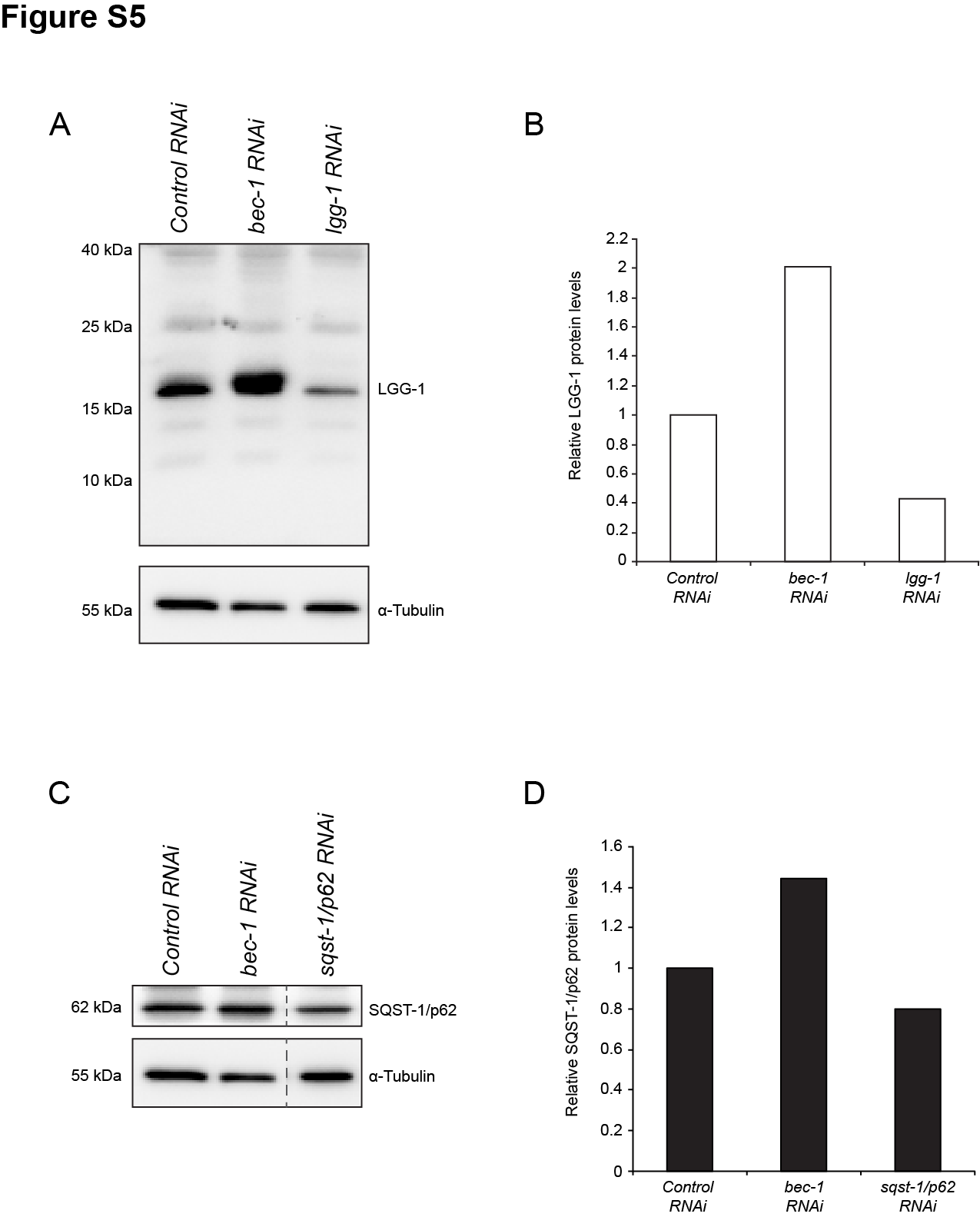

Supplement: Supplementary file 6 [file Image_5.TIF]

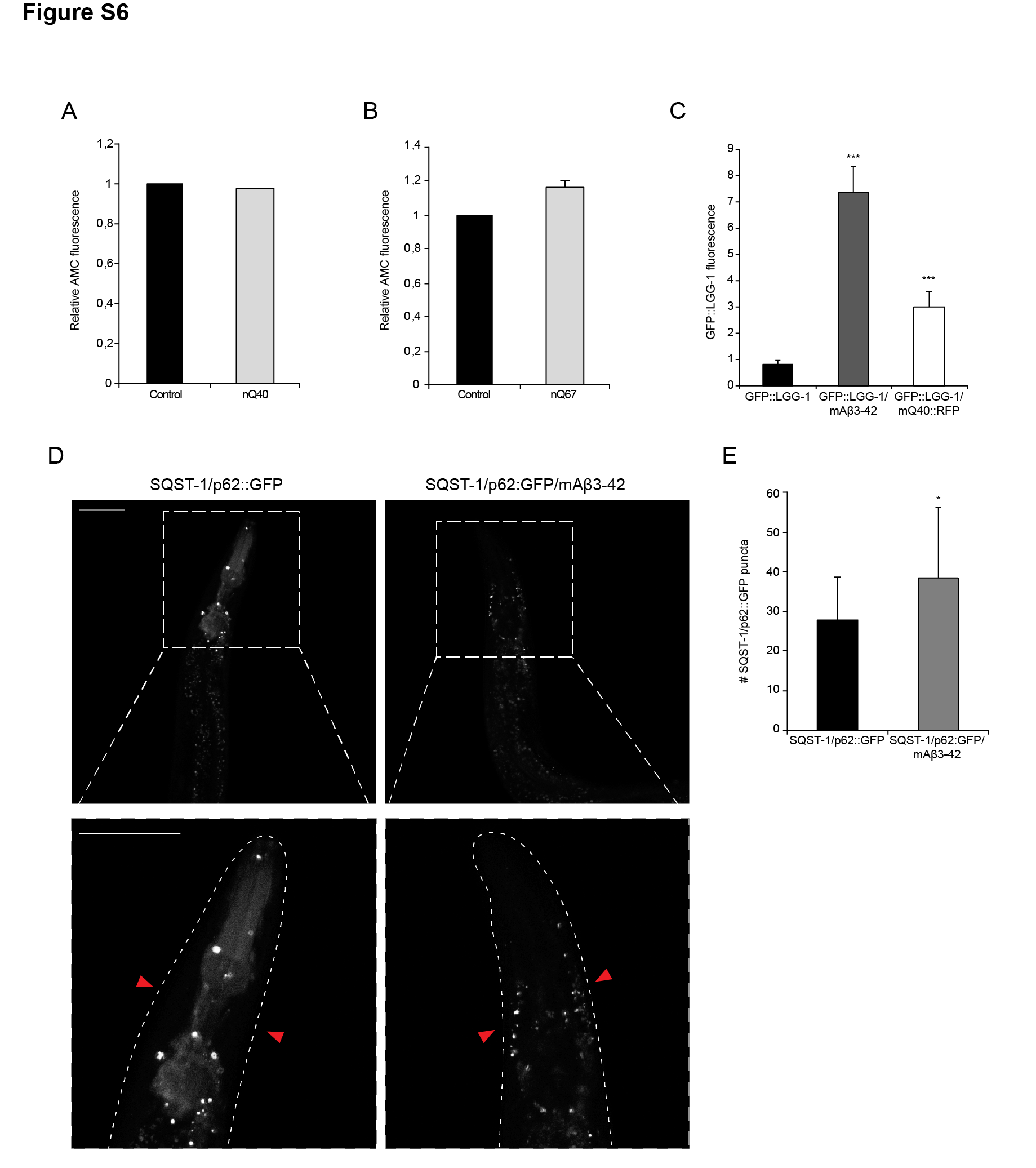

Supplement: Supplementary file 7 [file Image_6.TIF]

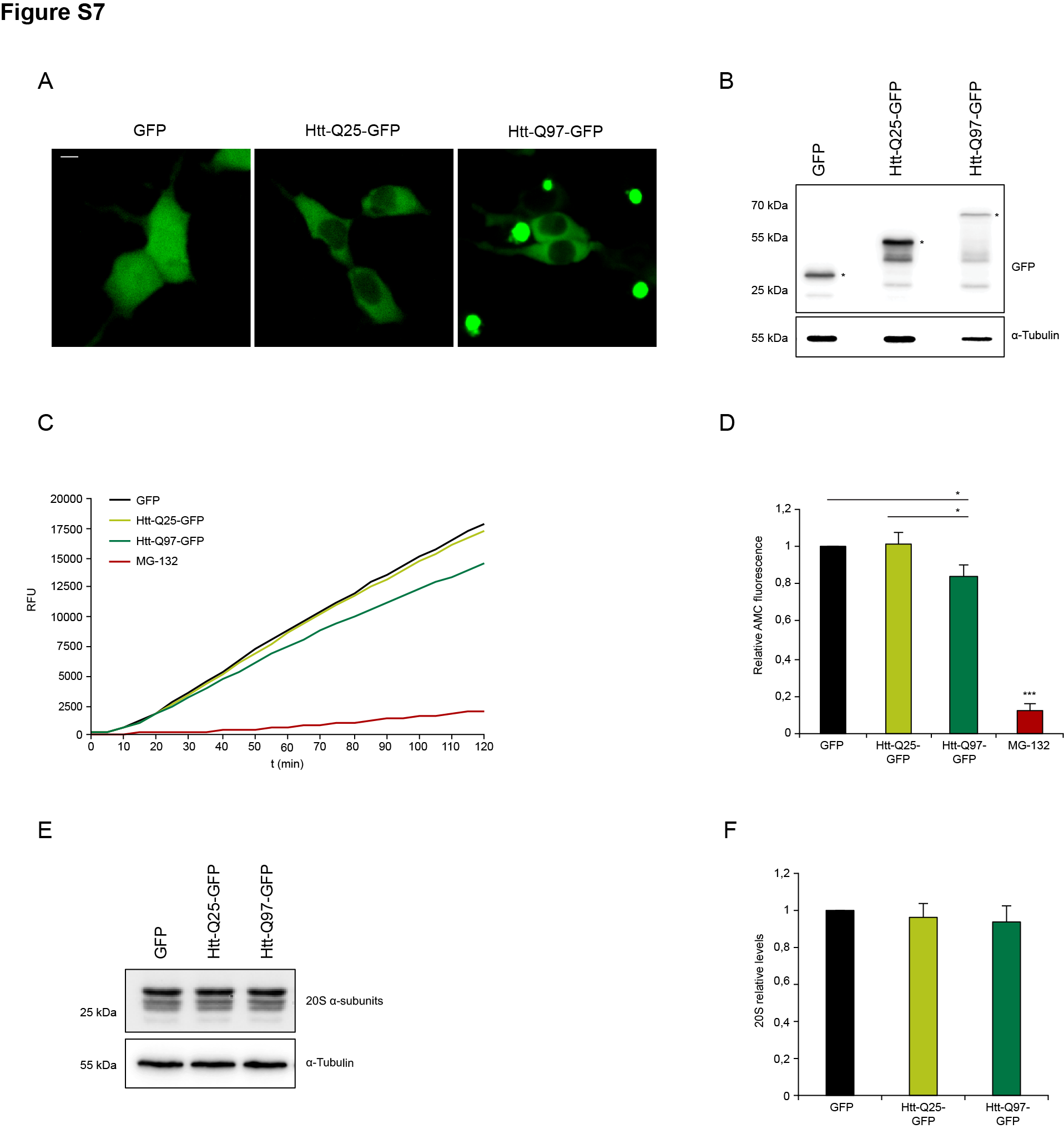

Supplement: Supplementary file 8 [file Image_7.TIF]

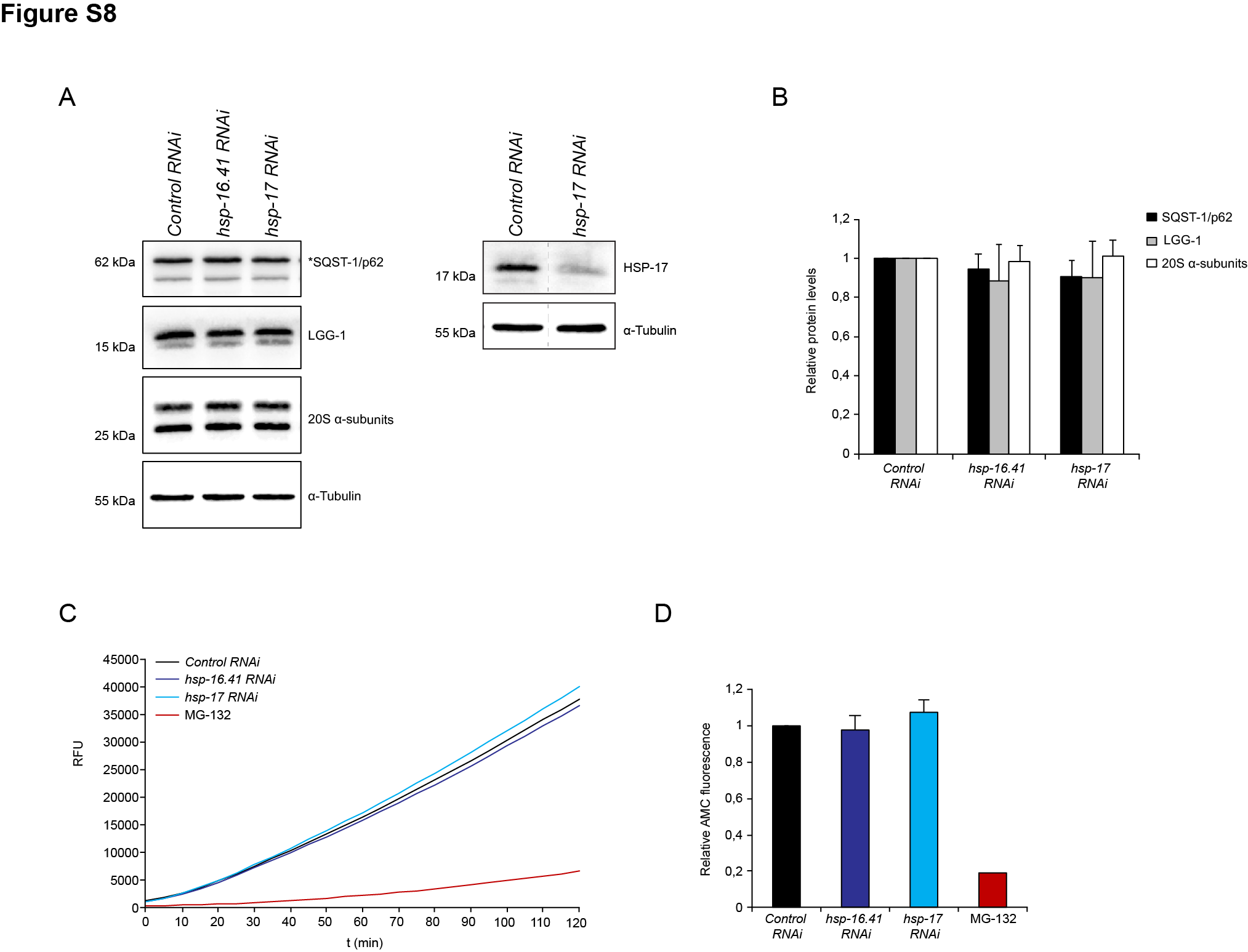

Supplement: Supplementary file 9 [file Image_8.TIF]
